# Cobalt isatin-Schiff-base derivative of MOF as a heterogeneous multifunctional bio-photocatalyst for sunlight-induced tandem air oxidation condensation process

**DOI:** 10.1038/s41598-023-32241-z

**Published:** 2023-03-29

**Authors:** Majid Rouzifar, Sara Sobhani, Alireza Farrokhi, José Miguel Sansano

**Affiliations:** 1 Department of Chemistry, College of Sciences, University of Birjand, Birjand, Iran; 2 Departamento de Química Orgánica, Facultad de Ciencias, Centro de Innovación en Química Avanzada (ORFEO-CINQA), Universidad de Alicante, Apdo. 99, 03080 Alicante, Spain

**Keywords:** Chemistry, Organic chemistry, Synthetic chemistry methodology

## Abstract

A sunlight-induced tandem air oxidation-condensation of alcohols with *ortho*-substituted anilines or malononitrile for the efficient synthesis of benz-imidazoles/-oxazoles/-thiazoles, or benzylidene malononitrile catalyzed by Co-isatin-Schiff-base-MIL-101(Fe) as a heterogeneous multifunctional bio-photocatalyst is reported. In these reactions, Co-isatin-Schiff-base-MIL-101(Fe) acts both as a photocatalyst, and a Lewis acid to catalyze the reaction of the in-situ formed aldehydes with *o*-substituted anilines or malononitrile. A significant decrease in the band gap energy and an increase in the characteristic emission of MIL-101(Fe) after functionalization with cobalt Schiff-base according to the DRS analysis and fluorescence spectrophotometry, respectively, indicate that the photocatalytic effectiveness of the catalyst is associated primarily to the synergetic influence of Fe–O cluster and Co-Schiff-base. EPR results obviously pointed out that Co-isatin-Schiff-base-MIL-101(Fe) is capable of creating ^1^O_2_ and O_2_^⋅−^ as active oxygen species under visible light irradiation. Using an inexpensive catalyst, sunlight irradiation, air as a cost-effective and abundant oxidant, and a low amount of the catalyst with recoverability and durability in ethanol as a green solvent, make this methodology as an environmentally friendly process with energy-saving organic synthetic strategies. Furthermore, Co-isatin-Schiff-base-MIL-101(Fe) displays excellent photocatalytic antibacterial activity under sunlight irradiation against *E. coli, S. aureus* and *S. pyogenes.* Based on our knowledge, this is the first report of using a bio-photocatalyst for the synthesis of the target molecules.

## Introduction

Currently, from the sustainable chemistry standpoint, decreasing environmental contamination and rising energy deficiency, are progressively concerned^1^. Tandem reactions which contain two or more consecutive independent reactions implemented in a one-pot manner with no isolation and purification of the intermediates, diminish the number of reaction steps, the solvent and reagents usage and consequently decrease the waste production and energy consumption.

Development of simple methods for the synthesis of required organic intermediates or final products from readily available reagents constitutes a key challenge in the synthetic organic chemistry^2,3^. Alcohols are considered as the most environmentally benign compounds, because of the readily availability, low cost, chance of being produced from renewable biomass resources, less poisoning, and ease of treatment, storage and transportation^4^. Therefore, the synthesis of organic compounds directly from alcohols by tandem organic process (TOP) has been known as an environmentally friendly chemical synthetic method^5^.

Although traditional thermal routs still have a significant role in current chemistry, a number of subsequent problems like waste byproducts formation due to some undesirable thermal side reactions urge to move towards using a renewable energy source^6,7^. In this regard, mimicking the nature in the consumption of sunlight to yield nutrient and oxygen in the plants, leads chemists to employ this available, free-charge and renewable energy source for the activation of catalytic transformations^8^. The examples of using solar energy as the activator in the photocatalytic transformations involve the removal of drugs^9^, organic dyes and heavy metal ions from wastewater by photo-degradation or -reduction^10^, inhibition of air contaminants, hydrogen generation by water splitting as well as chemical synthesis^11^. Primary studies in this field involved the use of photocatalysts with wide band gap like titanium or zinc oxide^12,13^. However, the big band gap and the fast recombination of electron–hole pairs cause low photocatalytic productivity and poor practicality. Moreover, the structure of traditional semiconductors, which are not matched, restricted their extra progress in the field of photocatalysis. Therefore, the discovering of efficient photocatalysts to substitute the conventional ones is highly demanded^14^.

Metal–organic frameworks (MOFs), a hybrid material with a three-dimensional crystalline structure, are discovering as a distinctive type of heterogeneous photocatalysts, due to their admirable photoexcitation and charge transition mechanism^15–18^. The inherent light absorption of MOFs can be tuned by changing either the metal ions as network nodes or the conjugation amount in the organic ligands^12,19–21^. In recent years, the use of MOFs for light-initiated oxidation reactions such as oxidation of saturated carbon-hydrogen bonds, hydroxylation of benzene, and selective oxidation of sulfides has been extensively duccumented^22–24^. Furthermore, due to the individual structure of MOFs which contains diverse catalytic sites such as metal nodes, ligands and catalytic species encapsulated in the holes, they have been used as multifunctional catalysts in tandem reactions such as epoxidation-ring opening, Sonogashira Hagiwara-click and olefination-hydrogenation reactions^25–27^. However, the use of MOF-based materials as multifunctional catalysts under light irradiation for the TOP reactions starting from alcohols are in the infancy stages^28–34^.

In continue of our studies on the introducing new heterogeneous catalysts for the organic transformations^35–37^, herein, in this paper, we have applied a MOF modified with cobalt-complex^38^ [Co-isatin-Schiff-base-MIL-101(Fe)] as a new heterogeneous multifunctional bio-photocatalyst for the synthesis of benz-imidazoles/-oxazoles/-thiazoles, and benzylidene malononitrile by TOP starting from readily available alcohols. The photocatalytic antibacterial activity of Co-isatin-Schiff-base-MIL-101(Fe) against *E. coli, S. aureus* and *S. pyogenes* was also studied under sunlight illumination.

## Experimental

### General information

Chemicals were purchased from Merck Company. The reactions were monitored by TLC using silica-gel polygram SILG/UV254 plates. X-ray photoelectron spectroscopy (XPS) was done by a VG-Microtech Multilab 3000 spectrometer, equipped with an Al anode. The deconvolution of spectra was done by Gaussian Lorentzian curves. X-ray powder diffraction (XRD) was carried out on an Xpert Pro Panalytical diffractometer incorporating Cu Kα radiation (λ = 0.154 Å). TEM images were obtained by a TEM microscope (Philips CM30). Field emission scanning electron microscopy (FESEM) images were obtained using a FESEM (model Mira 3-XMU). UV–Vis diffuse reflectance spectroscopy (UV–Vis-DRS, Shimadzu Co., Japan), FT-IR spectra were recorded on a Nicolet-Impact 400D spectrometer in the range of 400–4000 cm^−1^. The cobalt catalyst percentage was measured using an OPTIMA 7300DV ICP analyzer. EPR data were achieved on a Bruker EMX (freq. 9.80 GHz, time 80 ms, modulation freq. 100 kHz). Experiments were performed under sunlight irradiation between 8.00 a.m. and 4.00 p.m. (July to August 2021, at ~ 30 °C).

### Synthesis of Fe-MIL-101-isatin-Schiff-base

MIL-101(Fe)-NH_2_ (0.13 g)^33^ was added to a solution of isatin (0.147 g, 1 mmol) in ethanol (20 mL) and then refluxed for 1 day. The brown solid was centrifuged, washed with EtOH (3 × 10 mL) and dried at 60 °C in a vacuum oven to give the brown isatin-Schiff-base-MIL-101(Fe).

### Synthesis of Co-isatin-Schiff-base-MIL-101(Fe)

Co(OAc)_2_⋅4H_2_O (0.05 g, 0.2 mmol) was added to a mixture of isatin-Schiff-base-MIL-101(Fe) (0.1 g) in ethanol (15 mL) and stirred under reflux conditions for 24 h. Then, the solid was centrifuged, washed with EtOH (3 × 10 mL) and dried in a vacuum oven. The total amount of cobalt per gram of Fe-MIL-101-isatin-Schiff-base-Co was found to be 2.24 mmol by ICP analysis.

### Typical method for photocatalytic oxidation of benzyl alcohol in the presence of Co-isatin-Schiff-base-MIL-101(Fe)

A mixture of catalyst (1 mol%), benzyl alcohol (1 mmol), and EtOH (6 mL) was stirred in a glass tube under sunlight irradiation and air bubbling at ~ 30 °C. The reaction progress followed by TLC. After an appropriate time (Table 3), the catalyst was isolated by centrifugation and washed with ethanol. The solvent of the combined organic phase was evaporated under reduced pressure. The pure product was obtained by column chromatography.

### General route for the synthesis of benz-imidazoles/oxazoles/thiazoles via Tandem photo-oxidation-condensation reaction from benzyl alcohols and *o*-phenylenediamines catalyzed by Co-isatin-Schiff-base-MIL-101(Fe)

A mixture of Co-isatin-Schiff-base-MIL-101(Fe) (1.2 mol %, 5.3 mg), benzyl alcohol (1 mmol) and *o*-phenylenediamine/*o*-aminophenol/*o*-aminothiophenol (1 mmol) in ethanol (6 mL) was stirred at ~ 30 °C, while exposed to the sunlight, and air was bubbled into the reaction mixture (1 mL min^−1^). The reaction progress followed by TLC. After an appropriate time (Table 5), the catalyst was isolated by centrifugation, washed with ethanol (3 × 5 mL), and then reused in subsequent runs. The yellow product was obtained after addition of ice water to the remaining solution. The precipitated product was filtered and recrystallized in ethanol to provide the pure product.

### Tandem photo-oxidation/Knoevenagel condensation reaction catalyzed by Co-isatin-Schiff-base-MIL-101(Fe)

Benzyl alcohol (1 mmol) and malononitrile (1 mmol) were added to a glass tube containing Co-isatin-Schiff-base-MIL-101(Fe) (1.5 mol%) and EtOH (6 mL) equipped with a stirring bar. The reaction mixture was stirred at ∼ 30 °C with mild air flow under sunlight irradiation. After appropriate times (Table 8), as monitored by TLC (elution: *n*-hexane/ethyl acetate: 10:1), the catalyst was separated by centrifugation. The solvent of the remaining solution was evaporated under vacuum to produce the crude product. The pure product was obtained by ethanol–water recrystallization.

## Results and discussion

### Synthesis and characterization of Co-isatin-Schiff-base-MIL-101(Fe)

**Figure 1:**
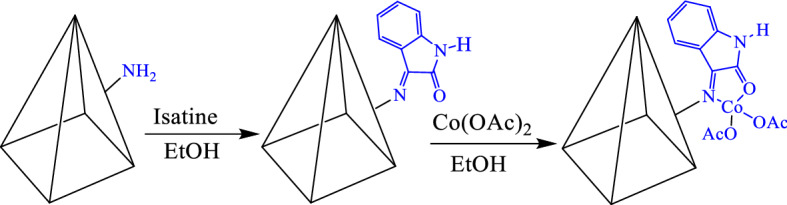
Synthetic rout for the preparation of Co-isatin-Schiff-base-MIL-101(Fe).

Co-isatin-Schiff-base-MIL-101(Fe)^38^ was synthesized via post modification method (PSM) of MIL-101(Fe)-NH_2_ (Fig. 1).

**Figure 2:**
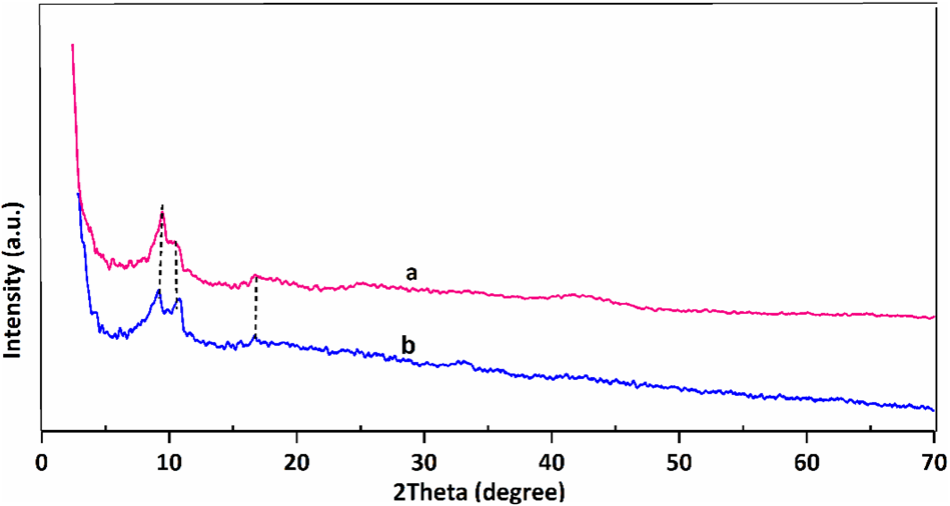
PXRD patterns of (a) MIL-101(Fe)–NH_2_ and (b) Co-isatin-Schiff-base-MIL-101(Fe).

Figure 2 shows the PXRD pattern of the as-synthesized MIL-101(Fe)–NH_2_ and the Co-isatin-Schiff-base-MIL-101(Fe). The well-defined diffraction peaks at 2θ of 9.2, 10.5, and 16.6º have revealed the high crystallinity of Fe-MIL-101-NH_2_. The XRD pattern of Fe-MIL-101-isatin-Schiff-base-Co is shown diffraction peaks at 2θ of 9.2, 10.6, and 16.6º. The slight changes observed in the XRD of the functionalized sample compared to the original MOF are probably due to the creation of some defects in the catalyst structure during the functionalization reaction.

To further investigation of the presence and types of the elements expected for the Co-isatin-Schiff-base-MIL-101(Fe), X-Ray photoelectron spectroscopy (XPS) was performed as depicted in Fig. S1. C 1s spectrum represents four peaks corresponded to C=C (284.4 eV), C=N (285.4 eV), C–N (287.5 eV) and C=O (289.2 eV) (Fig. S1a)^39^. The N 1s spectrum (Fig. S1b) consists of peaks at 398.5 and 400.6 eV, which are related to C=N and N–H bonds, respectively^40^. Two peaks at 711.3 eV and 724.9 eV (Fig. S1c) for Fe 2p_3/2_ and Fe 2p_1/2_, respectively, confirm the existence of Fe^+3^ in the MOF^41^. Presence of two pairs of distinct peaks in the high resolution XPS of Co 2p (Fig. S1d) at 781.3 (Co 2p_3/2_) and 797.3 eV (Co 2p_1/2_), indicates the existence of Co^2+^ in Co-isatin-Schiff-base-MIL-101(Fe)^42,43^.

**Figure 3:**
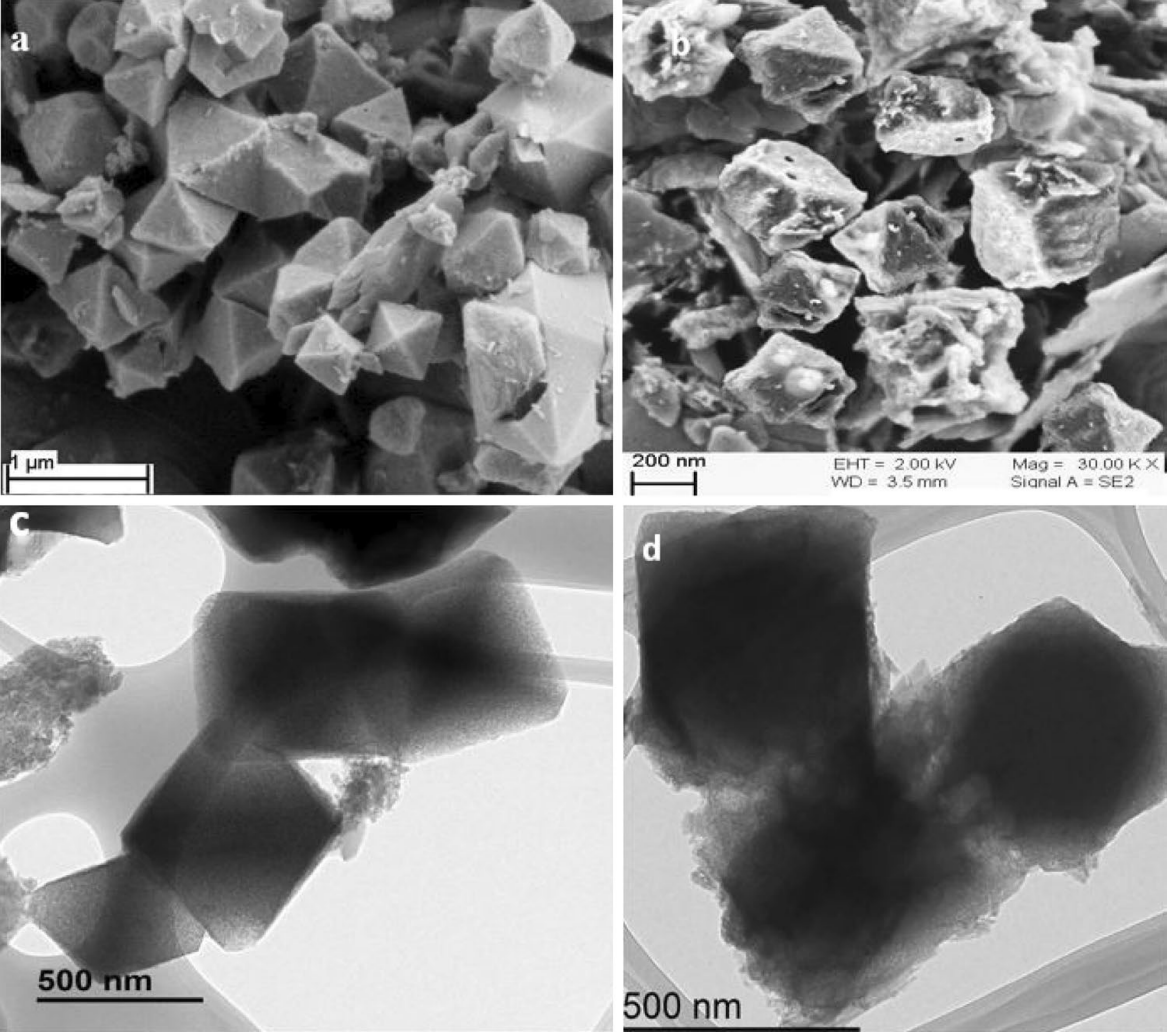
FESEM images of (**a**) MIL-101(Fe)–NH_2_ and (**b**) Co-isatin-Schiff-base-MIL-101(Fe); TEM images of (**c**) MIL-101(Fe)–NH_2_ and (**d**) Co-isatin-Schiff-base-MIL-101(Fe).

Morphology of Co-isatin-Schiff-base-MIL-101(Fe) and MIL-101(Fe)–NH_2_ were established by field emission scanning electron microscopy and transmission electron microscopy (FESEM and TEM). As shown in Fig. 3, the morphology of MIL-101(Fe)–NH_2_ remains nearly intact after PSM.

**Figure 4:**
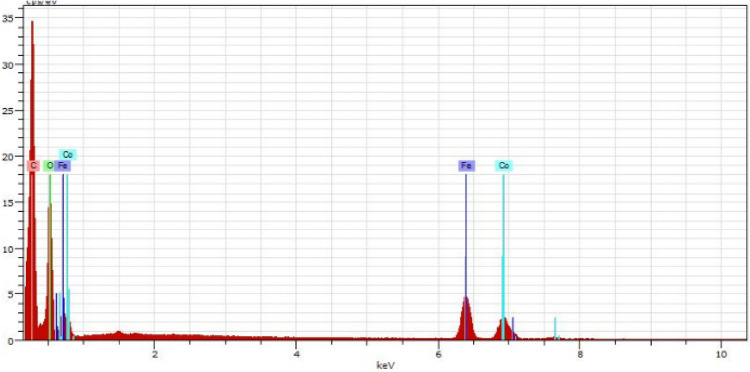
EDX spectrum of Co-isatin-Schiff-base-MIL-101(Fe).

**Figure 5:**
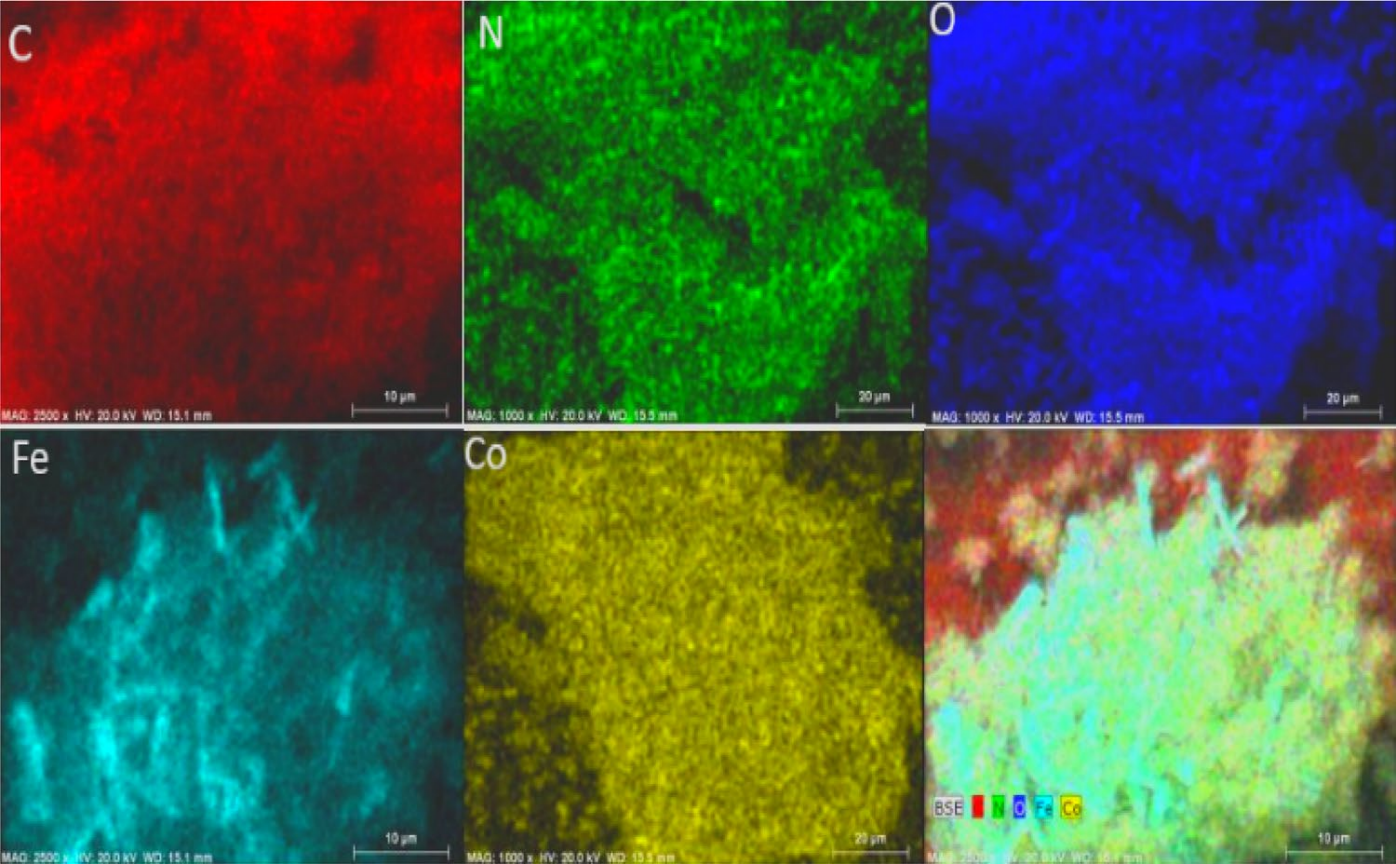
EDX mapping images of Co-isatin-Schiff-base-MIL-101(Fe).

Based on EDX spectrum, elements including carbon, oxygen, nitrogen, iron and cobalt present in Co-isatin-Schiff-base-MIL-101(Fe) (Fig. 4). Investigation of EDX-mapping indicates a uniform distribution of these elements in the catalyst (Fig. 5).

**Figure 6:**
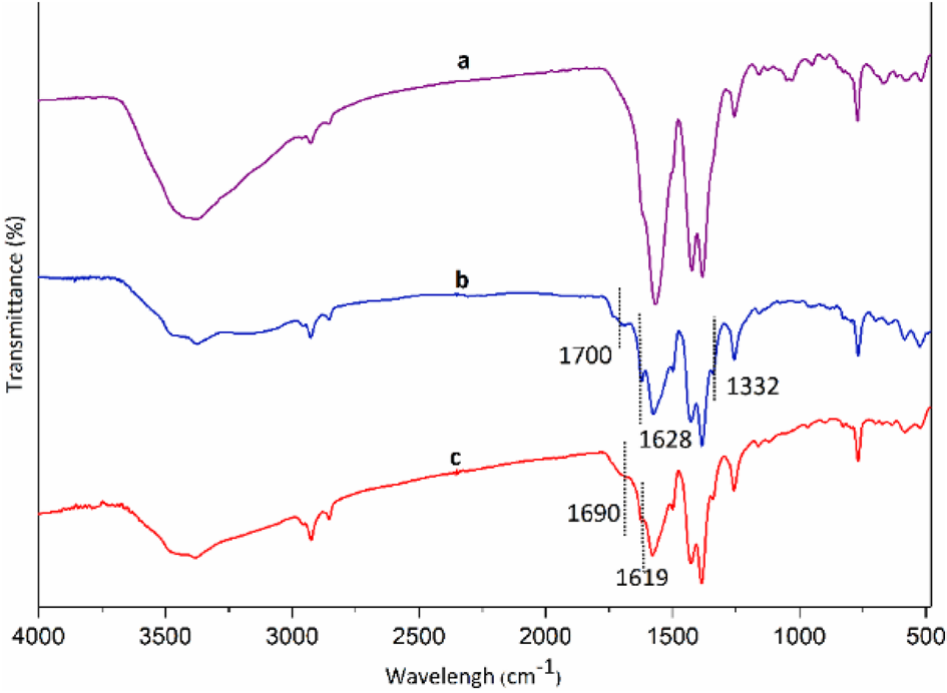
FT-IR spectra of (a) MIL-101(Fe)–NH_2_, (b) isatin-Schiff-base-MIL-101(Fe) and (c) Co-isatin-Schiff-base-MIL-101(Fe).

Study of the FT-IR spectra of the as-synthesized MIL-101(Fe)–NH_2_ and Co-isatin-Schiff-base-MIL-101(Fe) (Fig. 6a,c) ensured the successful construction of the targeting MOF. In the spectrum of MIL-101(Fe)–NH_2_, the peaks at 1382–1430 (C=C) and 1501–1600 (C=O) cm^−1^ are attributed to the 2-aminoterephthalate linkers (Fig. 6a). After PSM of MIL-101(Fe)–NH_2_, some new peaks at around 1700 (C=O), 1628 (C=N) and 1332 (C–N) cm^−1^ are formed, which could be attributed to the isatin-Schiff-base (Fig. 6b). Complex formation of isatin-Schiff-base in the isatin-Schiff-base-MIL-101(Fe) with cobalt is associated with the shift of vibration frequencies of C=O and C=N groups to the lower amounts (Fig. 6c).

Comparison of the N_2_ adsorption–desorption of Co-isatin-Schiff-base-MIL-101(Fe) with MIL-101(Fe)–NH_2_ (Fig. S2a,b) showed that the surface areas and total pore volumes are considerably reduced in Co-isatin-Schiff-base-MIL-101(Fe) (Table 1). The pore size distribution of the two samples is nearly identical (Fig. S2c,d). These results indicated the successful incorporation of Co (II) complexes into MIL-101(Fe)-NH_2_ with uniform distribution.

**Table 1:** Textural properties of pure MIL-101(Fe)–NH_2_ and Co-isatin-Schiff-base-MIL-101(Fe).

Sample	S_BET_^a^ (m^2^ g^−1^)	V_total_^b^ (cm^3^ g^−1^)
MIL-101(Fe)–NH_2_	1810	1.08
Co-isatin-Schiff-base-MIL-101(Fe)	295	0.25

^a^BET surface area.

^b^Total pore volume.

**Figure 7:**
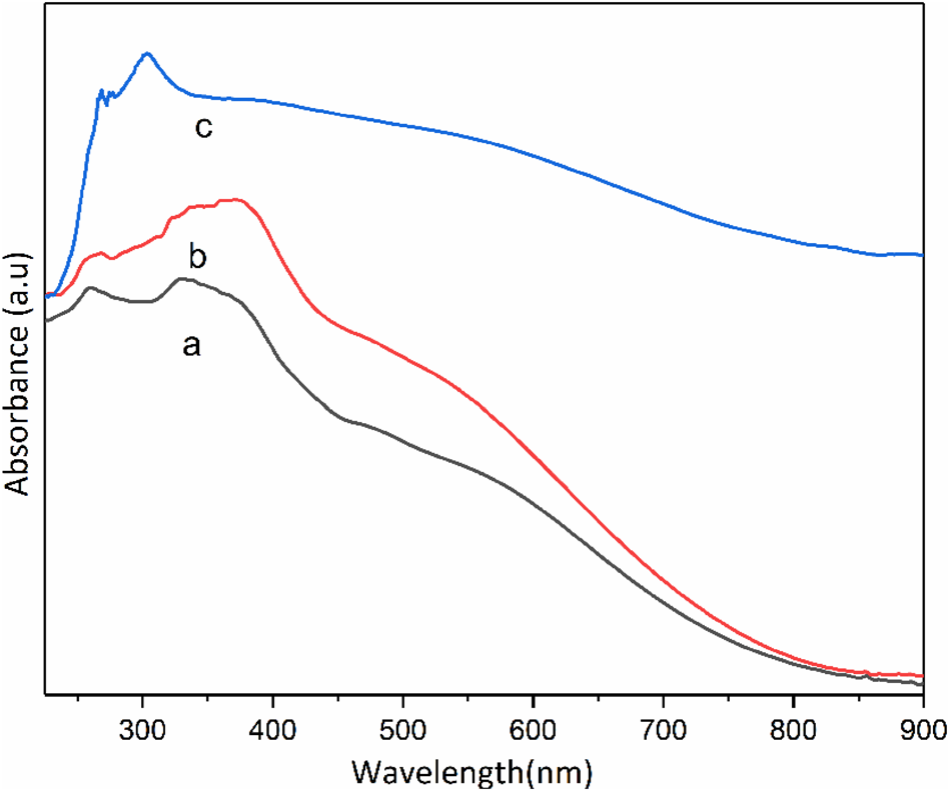
UV–visible absorption spectra of (a) MIL-101(Fe)-NH_2_, (b) isatin-Schiff-base-MIL-101(Fe) and (c) Co-isatin-Schiff-base-MIL-101(Fe).

**Figure 8:**
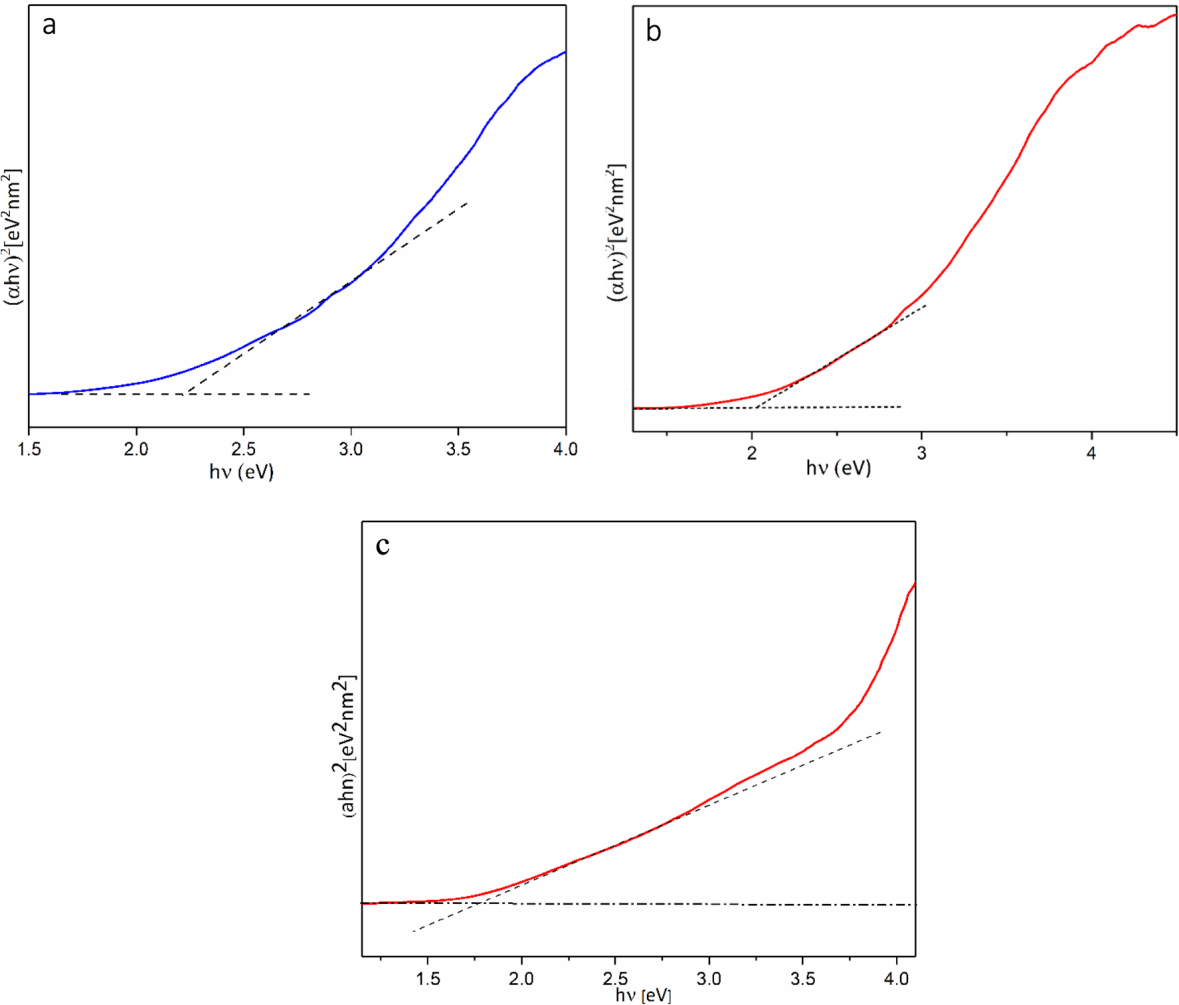
Band gap energy of (**a**) MIL-101(Fe)-NH_2_, (**b**) isatin-Schiff-base-MIL-101(Fe) and (**c**) Co-isatin-Schiff-base-MIL-101(Fe).

The UV–Vis DRS spectrum of Co-isatin-Schiff-base-MIL-101(Fe) shows a broad absorption band in the visible light region (250–800 nm) compared with MIL-101(Fe)–NH_2_ and isatin-Schiff-base-MIL-101(Fe) (Fig. 7). These results showed that Co-isatin-Schiff-base-MIL-101(Fe) can be excited in the visible region, due to the existence of Fe–O clusters in conjunction with cobalt Schiff-base, which facilities the π–π transitions. The DRS analysis showed a significant decrease in the band gap energy (Eg) of MIL-101(Fe)–NH_2_ from 2.23^44^ to 2.03 eV after modification by isatin and 1.7 eV after complex formation with cobalt (Fig. 8).

### Synthesis of benz-imidazoles/-oxazoles/-thiazoles from alcohols via TOP catalyzed by Co-isatin-Schiff-base-MIL-101(Fe)

**Figure 9:**
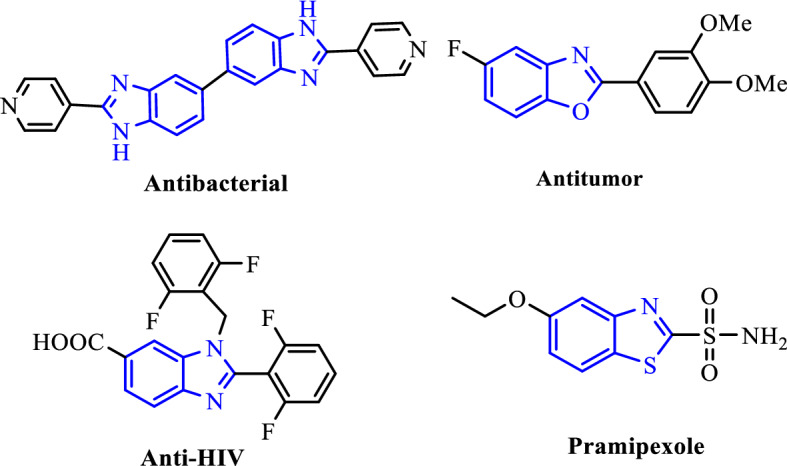
Examples of biologically and pharmaceutically relevant benz-imidazoles/-oxazoles/-thiazoles.

2-Substituted benz-imidazoles/-oxazoles/-thiazoles as significant types of heterocycles, have been broadly documented as structural blocks in the preparation of biological and pharmaceutical compounds (Fig. 9)^45–47^.

The methods for the synthesis of these heterocyclic compounds consist of the reaction of *o*-phenylenediamine with a variety of reagents. Acids, aldehydes, esters, anhydrides, amides, acyl halides, and nitriles are the common reagents that have been introduced in these methods^48–50^. However, their synthesis directly from readily available alcohols by TOP is more desirable. In these reactions, aldehydes play as highly active reaction intermediates to undergo the subsequent reactions such as condensation with *o*-phenylene diamine, *o-*aminophenol, and/or *o-*aminothiophenol. Numerous materials have been reported to activate these reactions such as porous catalysts, ionic liquids, metal oxides, graphene oxide and MOFs^51–57^. The reported methods associated with drawbacks such as requiring long reaction time, high temperature, low yields of the products, use of expensive/toxic metals, unrecoverable catalyst and poisonous solvents that may cause environmental pollution^58–60^.

Therefore, the development of highly efficient alternate methods which proceed under economic and environmental-friendly conditions with energy-saving organic synthetic strategies is desirable. In this paper, we have studied the application of Co-isatin-Schiff-base-MIL-101(Fe) as a heterogeneous multifunctional bio-photocatalyst for the synthesis of benz-imidazoles/oxazoles/thiazoles via TOP starting from alcohols. Based on our knowledge, this is the first report of using a bio-photocatalyst for the synthesis of these target molecules.

Initially, the catalyst efficiency for the alcohol photo-oxidation was investigated by selecting benzyl alcohol as a typical compound and the effect of different catalyst loading, solvents, light source and the oxidizing agent was tested for the oxidation of this compound (Table 2). Satisfied results were obtained when 1 mol% of Co-isatin-Schiff-base-MIL-101(Fe) was used in ethanol under sunlight irradiation and air bubbling. The effect of solvents with different polarities on the progress of the reaction was investigated (Table 2, entries 4–10). Low yield of the product under solvent-free conditions (Table 2, entry 10) showed the importance of the solvent for transferring the substrates into the active sites of the catalyst. The lowest yield was obtained in water (Table 2, entry 7) among other organic solvents and the highest yield was observed in ethanol (Table 2, entry 4). The increase in the efficiency of the catalytic process in ethanol can be due to the greater solubility of substances in ethanol and subsequently the easier transfer of substances to the MOF pores and as a result faster interaction with the active catalytic sites. Solvents such as DMF and THF can be coordinated to the active sites of the MOF catalyst and so decrease its catalytic efficiency. The model reaction proceeded with low yields in the presence of H_2_O_2_ or TBHP (Table 2, entries 16 and 17) probably due to the restricted access to the active sites of the catalyst and their different mechanism pathways. To find the effect of the light on the progress of the reaction, the model reaction was also studied under dark reaction conditions and benzaldehyde was obtained in an insignificant amount after 8 h (Table 2, entry 13). When a similar reaction was performed under N_2_ atmosphere, any conversion of benzyl alcohol was not observed (Table 2, entry 18).

**Table 2:** Optimization of photo-oxidation of benzyl alcohol to benzaldehyde.


Entry^a^	Catalyst (mol%)	Oxidant	Solvent	Time (h)	Yield (%)
1	0	Air	EtOH	4	0
2	0.4	Air	EtOH	5	73
3	0.7	Air	EtOH	4	85
4	1	Air	EtOH	4	90
5	1	Air	THF	4	60
6	1	Air	Toluene	4	65
7	1	Air	H_2_O	5	45
8	1	Air	CH_3_CN	6	86
9	1	Air	DMF	5	70
10	1	Air	–	4	55
11^b^	1	Air	EtOH	4	85
12^c^	1	Air	EtOH	4	70
13^d^	1	Air	EtOH	8	20
14^e^	1	–	EtOH	6	0
15	1	O_2_	EtOH	5	92
16f.	1	H_2_O_2_	EtOH	4	30
17f.	1	TBHP	EtOH	4	35
18^e^	1	–	EtOH	4	0

^a^Reaction conditions: benzyl alcohol (1 mmol), EtOH (6 mL), catalyst (1 mol%, except in entry 1), at ~ 30 °C, under air bubbling (except in entries 14–18) and sunlight irradiation (except in entries 11–13).

^b^Blue LED visible light irradiation.

^c^Room light.

^d^Without irradiation (dark).

^e^Under N_2_ atmosphere.

^f^TBHP and H_2_O_2_ (1 mmol).

To examine the generality of this photo-oxidation method, the reaction of a variety of alcohols was performed using optimized reaction conditions. As shown in Table 3, selective photo-oxidation of primary and secondary benzyl alcohols occurred and the corresponding carbonyl compounds were obtained in 78–90% yields without any overoxidation (entries 1–8). Furfuryl alcohol, as a famous challenging heteroaromatic alcohol and cinnamyl alcohol, containing a conjugated double bond, underwent selective photo-oxidation to furfural and cinnamaldehyde, respectively (Table 3, entries 9 and 10). Aliphatic alcohols exhibited less activity using the present photo-oxidation method (Table 3, entries 11–14).

**Table 3:**
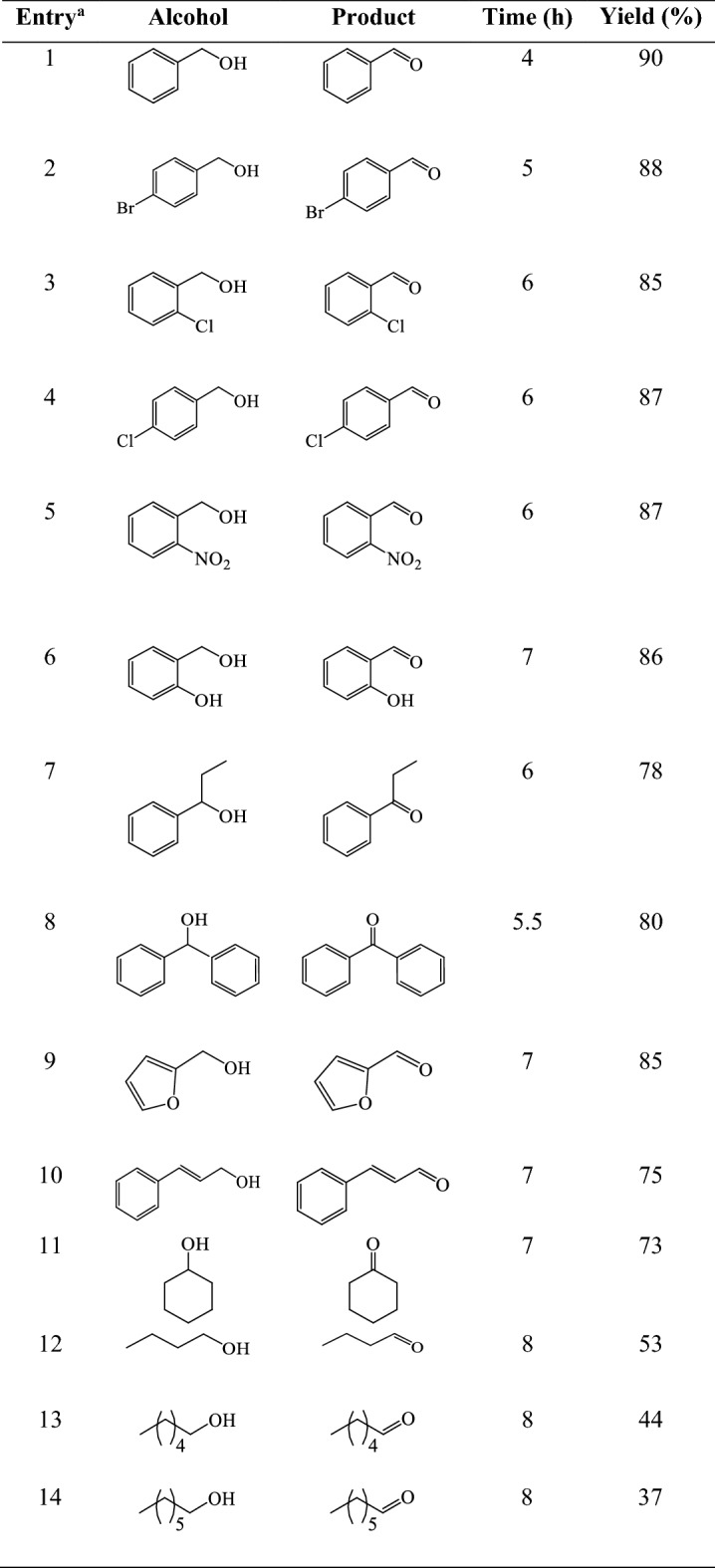
Photo-oxidation of alcohols catalyzed by Co-isatin-Schiff-base-MIL-101(Fe) in the presence of air under sunlight irradiation.

^a^Reaction conditions: alcohol (1 mmol), EtOH (6 mL), catalyst (1 mol%), ~ 30 °C, under air bubbling and sunlight conditions*.* Turnover number [TON (mol of the product per mol of the catalyst)] = 37–90, turnover frequency [TOF (TON/time)] = 4.6–22.5 ^–^h.

With successful photo-oxidation of alcohols, in the next part, the efficiency of Co-isatin-Schiff-base-MIL-101(Fe) was investigated in the synthesis of benzimidazoles from alcohols via TOP. The reaction of benzyl alcohol and *o-*phenylendiamine was chosen as a typical reaction to discover the best catalyst loading and the appropriate solvent (Table 4). The best result was achieved using 1.2 mol% of the catalyst in ethanol (Table 4, entry 6).

**Table 4:** Optimization of the reaction conditions for the synthesis of benzimidazoles from alcohols via TOP catalyzed by Co-isatin-Schiff-base-MIL-101(Fe).


Entry	Catalyst (mol %)	Solvent	Yield (%)
1	0	EtOH	0
2	0.2	EtOH	52
3	0.4	EtOH	66
4	0.7	EtOH	72
5	1	EtOH	83
6	1.2	EtOH	90
7	1.5	EtOH	90
8	1.2	THF	77
9	1.2	Toluene	74
10	1.2	H_2_O	29
11	1.2	DMF	78
12	1.2	CH_3_CN	85

Reaction conditions: *o*-phenylendiamine (1 mmol), benzyl alcohol (1 mmol), solvent (6 mL), 8 h, air bubbling and sunlight irradiation, ~ 30 °C.

The reactions of a number of benzyl alcohols containing electron-withdrawing or -releasing groups and *o*-substituted anilines catalyzed by Co-isatin-Schiff-base-MIL-101(Fe) were performed under the optimum reaction conditions (Table 5). As depicted in Table 5, benzyl alcohols and *o*-substituted anilines (*o*-phenylene diamine, *o*-aminophenol, and/or *o*-aminothiophenol) underwent the TOP reaction with high efficiency to give benz-imidazoles/-oxazoles/-thiazoles, respectively, in good to high yields. The functional groups (methyl, nitro, and chloride) in the benzyl alcohol stayed with no change in the TOP reaction.

**Table 5:** Photo-induced synthesis of different benz-imidazoles/-oxazoles/-thiazoles catalyzed by Co-isatin-Schiff-base-MIL-101(Fe) from alcohols via TOP.


Entry	Benzyl alcohol	X	Product	Yield (%)	Obtained M.P. (°C)	Reported M.P. (°C)^Ref^
1		NH		90	288–290	290–294^30^
2		NH		78	288–290	289–291^30^
3		NH		83	262–264	263–265^30^
4		NH		80	308–310	309–310^30^
5		O		83	198–200	201–203^30^
6		O		75	209–211	209–211^30^
7		O		80	90–92	88–90^58^
8		O		77	262–264	261–262^60^
9		S		80	109–110	110–112^30^
10		S		73	113–115	114–116^30^
11		S		76	84–86	85–87^61^
12		S		74	227–228	226–227^61^

Reaction conditions**:** *o***-**substituted anilines (1 mmol), benzyl alcohol (1 mmol), EtOH (6 mL), catalyst (1.2 mol%), 8 h, air bubbling, sunlight irradiation and ~ 30 °C. The products were identified by NMR spectroscopy (see supplementary information file). TON (mol of the product per mol of the catalyst = 61–75), TOF (TON/time) = 7.6–9.4 ^–^h.

**Figure 10:**
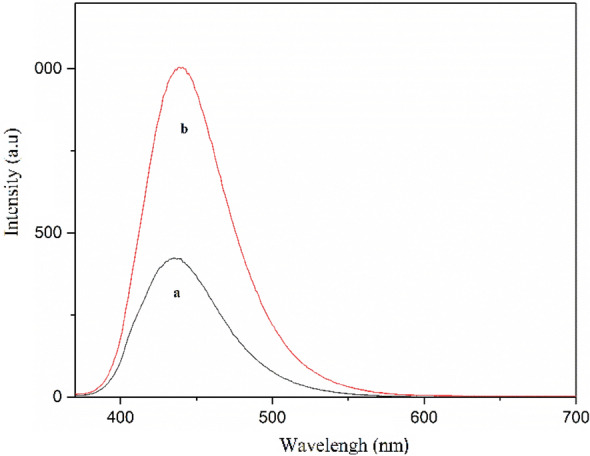
Emission spectra of (a) MIL-101(Fe)–NH_2_ and (b) Co-isatin-Schiff-base-MIL-101(Fe).

To understand the role of Co-isatin-Schiff-base-MIL-101(Fe) in this reaction, the photocatalytic efficiency of cobalt-free precursors of the catalyst [MIL-101(Fe)–NH_2_ and isatin-Schiff-base-MIL-101(Fe)] and Co-isatin-Schiff-base were examined in the model reaction for the synthesis of benzimidazole under the optimized reaction conditions (Table 6, entries 1–4). Comparison of the obtained results with those of Co-isatin-Schiff-base-MIL-101(Fe) showed a significant decrease in the catalytic efficiency of cobalt-free precursors and Co-isatin-Schiff-base. Similar reactions in the presence of FeCl_3_⋅6H_2_O or Co(OAc)_2_⋅4H_2_O did not show any significant progress (Table 6, entries 5 and 6). These experiments obviously revealed that the photocatalytic efficiency of Co-isatin-Schiff-base-MIL-101(Fe) is related primarily to the synergetic effect of Fe–O cluster and Co-Schiff-base. The presence of the synergetic effect could be clarified by a significant decrease in the band gap energy and an increase in the fluorescence emission of MIL-101(Fe) after functionalization with cobalt Schiff-base complex according to DRS analysis (Fig. 8) and fluorescence spectrophotometry (Fig. 10), respectively. In order to indicate the importance of sunlight irradiation and also air in the progress of the reaction, two more experiments were carried out. Under dark conditions, only a trace amount of benzimidazole was obtained (Table 6, entry 7) and benzyl alcohol remained intact in the presence of N_2_ atmosphere (Table 6, entry 8). Additionally, when benzaldehyde was used instead of benzyl alcohol as the staring material, the desired product was produced in shorter time (Table 6, entry 9). The effect of sunlight irradiation and also air in the progress of the reaction starting from benzaldehyde was studied. Under dark conditions or in the presence of N_2_ atmosphere, a low yield of benzimidazole was obtained (Table 6, entries 10 and 11). These experiments clearly indicate that the catalytic oxidation reaction takes place in two key steps: (1) selective photo-oxidation of benzyl alcohol to benzaldehyde and (2) dehydrogenation reaction, which takes place after condensation reaction of benzaldehyde with *o*-phenylendiamine.

**Table 6:** Effect of different catalytic species or conditions on the synthesis of benzimidazole.

Entry	Catalyst	Light irradiation	Yield (%)
1	MIL-101(Fe)–NH_2_	Sunlight	40
2	Isatin-Schiff-base-MIL-101(Fe)	Sunlight	50
3^a^	Co-isatin-Schiff-base	Sunlight	Trace
4	Co-isatin-Schiff-base-MIL-101(Fe)	Sunlight	90
5	FeCl_3_⋅6H_2_O	Sunlight	Trace
6	Co(OAc)_2_⋅4H_2_O	Sunlight	Trace
7	Co-isatin-Schiff-base-MIL-101(Fe)	Dark	Trace
8^b^	Co-isatin-Schiff-base-MIL-101(Fe)	Sunlight	Trace
9^c^	Co-isatin-Schiff-base-MIL-101(Fe)	Sunlight	90
10^c^	Co-isatin-Schiff-base-MIL-101(Fe)	Dark	25
11^c,b^	Co-isatin-Schiff-base-MIL-101(Fe)	Sunlight	25

Reaction conditions: o-phenylenaniline (1 mmol), benzyl alcohol (1 mmol, except in entries 9-11), EtOH (6 mL), catalyst (5.3 mg), 8 h (except in entry 9).

^a^Co-isatin-Schiff-base (isatin-Schiff-base was prepared by the reaction of isatin and aniline).

^b^Under N_2_ atmosphere.

^c^Benzaldehyde (1 mmol), 4 h.

**Figure 11:**
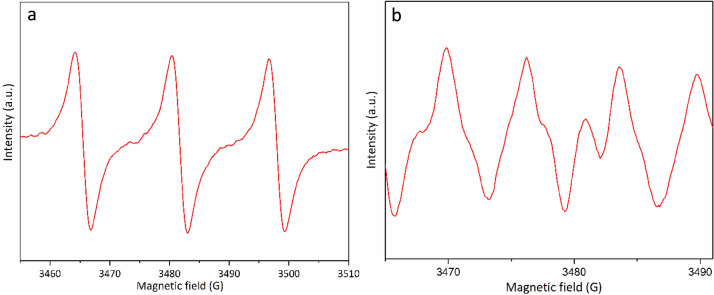
(**a**) EPR spectra for ^1^O_2_ detection by TEMP and (**b**) EPR detection of O_2_^⋅–^ by DMPO.

The electron paramagnetic resonance (EPR) measurements were recorded to detect reactive oxygen species (ROS) formed during the photocatalytic step. By using 2,2,6,6-tetramethylpiperidine (TEMP), as the singlet oxygen (^1^O_2_) detection agent, a strong 1:1:1 triplet signal was depicted (Fig. 11a), proving ^1^O_2_ formation over Co-isatin-Schiff-base-MIL-101(Fe) under the optimum conditions. When 5,5-dimethyl-1-pyrroline *N*-oxide (DMPO) as a superoxide radical anion (O_2_^⋅−^) detection agent was used, strong signals appeared (Fig. 11b), which indicates that O_2_^⋅−^ species were also formed during the photocatalytic reaction.

**Figure 12:**
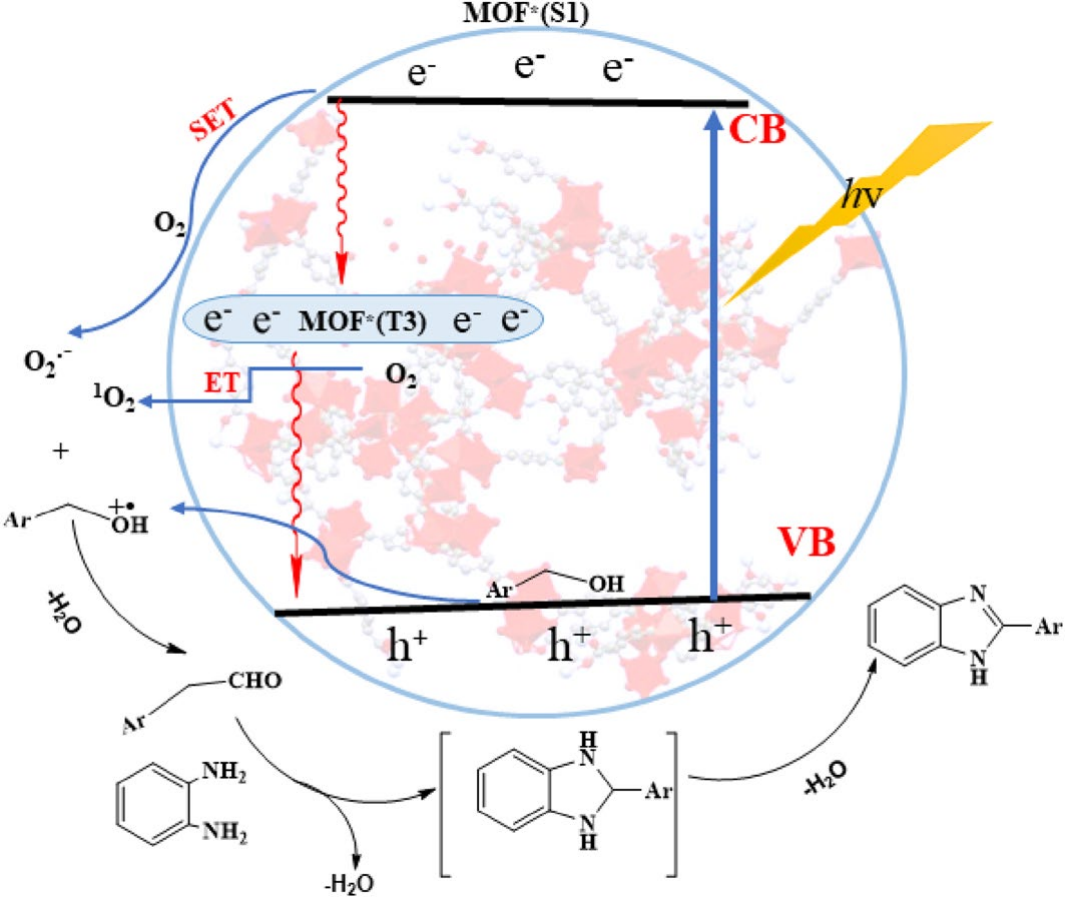
Proposed mechanism for the synthesis of 2-phenylbenzimidazole from *o*-phenylenediamine and alcohol over Co-isatin-Schiff-base-MIL-101(Fe).

Based on our observation and also the postulated mechanisms in the literature^28,29,34,62^, a mechanism is proposed in Fig. 12. When Co-isatin-Schiff-base-MIL-101(Fe) is irradiated, the singlet excited state of MOF and the holes were produced. The excited electrons in the valance band reduces O_2_ to form O_2_^⋅–^ by single electron transfer (SET). Moreover, singlet excited state of MOF can be converted to triplet excited state by intersystem crossing. The produced triplet excited state activates oxygen to ^1^O_2_ through energy transfer process. The holes (h^+^) oxidize benzyl alcohol into benzyl alcohol^⋅+^, which further reacted with formed O_2_^⋅−^ and ^1^O_2_ to produce benzaldehyde. In the next step, the Lewis acidic iron and cobalt sites in Co-isatin-Schiff-base-MIL-101(Fe) catalyze the condensation of the as-formed aldehyde and *o-*phenylenediamine to generate the cyclic compound, which is more oxidized to benzimidazole. To determine the active species involved in the catalytic cycle, the photocatalytic reaction was individually investigated using some famous scavengers. In the presence of *p*-benzoquinone (BQ), NaN_3_ and ammonium oxalate, the photocatalytic efficiency of the MOF was decreased obviously. The above results indicate that O_2_^⋅–^, ^1^O_2_ and h^+^ are the principal oxidants in the photocatalytic procedure.

**Figure 13:**
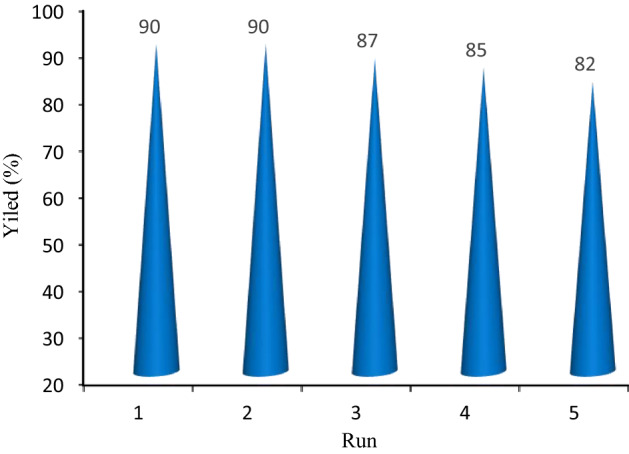
Reusability of the Co-isatin-Schiff-base-MIL-101(Fe) during synthesis of benzimidazole.

**Figure 14:**
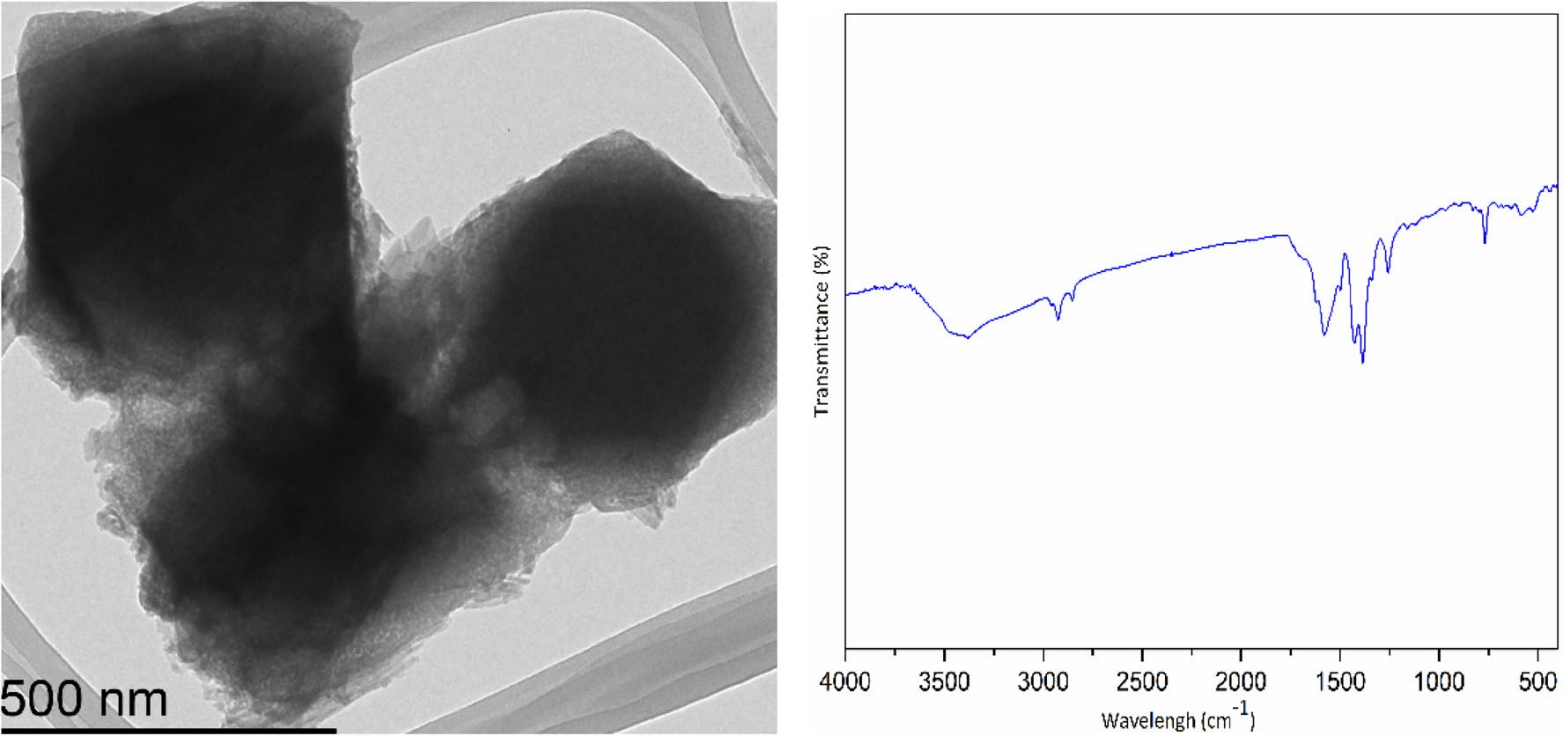
TEM image and FT-IR spectrum of Co-isatin-Schiff-base-MIL-101(Fe) after five times reuse in the synthesis of benzimidazole.

The reusability of Co-isatin-Schiff-base-MIL-101(Fe) was studied in the model reaction of the synthesis of benzimidazole from alcohol via TOP. For this purpose, after completion of the reaction, ethanol (10 mL) was added to the mixture and the reaction mixture was centrifuged to separate the catalyst. The recovered catalyst after washing with ethanol was reused in five consecutive runs in the model reaction with insignificant decrease in the catalytic activity (Fig. 13). The FT-IR spectrum, and TEM image of the reused catalyst indicate that the structure of the catalyst preserved after five times recycling (Fig. 14).

**Figure 15:**
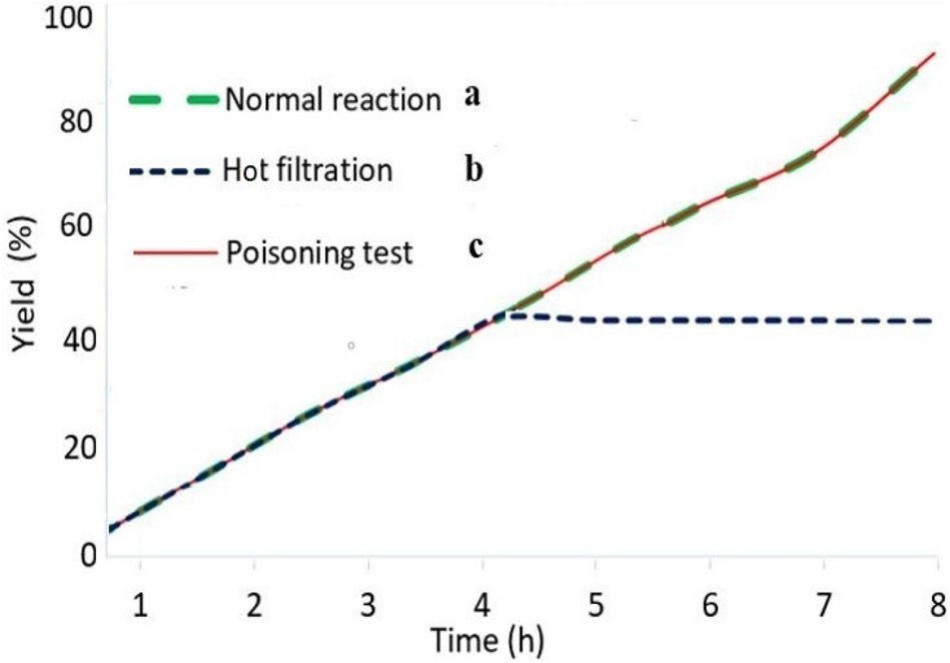
Synthesis of benzimidazole starting from alcohol using Co-isatin-Schiff-base-MIL-101(Fe) as a photocatalyst: (a) normal reaction, (b) employing a hot filtration protocol and (c) using S_8_ as a poison for the catalyst.

A classical hot-filtration examination was employed to explore whether Co-isatin-Schiff-base-MIL-101(Fe) works as a heterogeneous bio-photocatalyst or releases cobalt ions. Towards this point, synthesis of benzimidazole directly from alcohol was investigated under the optimized reaction conditions. In half time of the reaction (4 h), the reaction mixture was centrifuged to separate the catalyst. Then the reaction was allowed to be continued for an additional 4 h. Any progress in the reaction was not observed by increasing the reaction time (Fig. 15b). The nature of the catalytic mode (homogeneous or heterogeneous) was surveyed using a poisoning test. For this purpose, the model reaction of the synthesis of benzimidazole was performed in the presence of S_8_ (0.05 g) to kidnap the possible released cobalt species. No changes in the reaction course compared to normal one (Fig. 15a) were identified, which revealed the absence of the leached homogeneous cobalt particles in the solution (Fig. 15c).

### Tandem photo-oxidation/Knoevenagel condensation reaction catalyzed by Co-isatin-Schiff-base-MIL-101(Fe)

The results of benzimidazole synthesis from alcohols via TOP, encouraged us to investigate the photocatalytic performance of Co-isatin-Schiff-base-MIL-101(Fe) in the tandem photo-oxidation/Knoevenagel condensation reaction of alcohols and malononitrile. For this purpose, the reaction of benzyl alcohol and malononitrile under sunlight irradiation at ~ 30 °C was selected as a model reaction to optimize the reaction conditions such as the amount of the catalyst and solvent (Table 7). 1.5 mol% of Co-isatin-Schiff-base-MIL-101(Fe) was the most effective amount of the catalyst (Table 7, entry 3). Some solvents such as EtOH, THF, toluene, H_2_O, CH_3_CN and also solvent-free conditions were tested (Table 7, entries 3 and 5–10) and found that the reaction is efficiently promoted in EtOH compared to other solvents (Table 7, entry 3). To show the effect of the irradiation on the progress of the reaction, a similar reaction in the absence of any light and air was also studied and any conversion of the starting material was not detected (Table 7, entry 11).

**Table 7:** Tandem one-pot photo-oxidation/Knoevenagel condensation reaction of alcohols catalyzed by Co-isatin-Schiff-base-MIL-101(Fe).


Entry^a^	Catalyst	Solvent	Time (h)	Yield (%)
1	0.5	EtOH	8	67
2	0.9	EtOH	8	78
3	1.5	EtOH	5	88
4	2	EtOH	5	88
5	1.5	THF	5	50
6	1.5	Toluene	5	43
7	1.5	H_2_O	5	35
8	1.5	CH_3_CN	5	82
9	0	EtOH	5	trace
10	1.5	-	8	47
11^b,c^	1.5	EtOH	10	0

^a^Reaction conditions: benzyl alcohol (1 mmol), malononitrile (1 mmol), solvent (6 mL), catalyst (1.5 mol%), air bubbling, sunlight irradiation (except in entry 11), ~ 30 °C.

^b^Dark conditions.

^c^Under N_2_ atmosphere.

Having the optimized reaction conditions, the photo-oxidation/Knoevenagel condensation reaction of various alcohols were examined (Table 8). As the results of Table 8 show, benzyl alcohols containing different substituents underwent the photo-oxidation/Knoevenagel condensation reaction with malononitrile and produced the desired products in good to high yields (Table 8, entries 1–8). The reaction of ethyl cyanoacetate as a compound with active methylene and alcohol proceeded well under the same reaction conditions (Table 8, entries 9 and 10).

**Table 8:** TOP photo-oxidation/Knoevenagel condensation reaction of alcohols catalyzed by Co-isatin-Schiff-base-MIL-101(Fe).


Entry	R	X	Product	Time (h)	Yield (%)	Obtained M.P. (ºC)	Reported M.P. (ºC)^Ref^
1	H	CN		5	88	81–83	80–82^63^
2	CH_3_	CN		8	87	131–133	130^64^
3	OCH_3_	CN		8	88	113–114	111–113^63^
4	Br	CN		6	85	153–155	152–154^63^
5	Cl	CN		6.5	83	163–165	162–163^63^
6	2-Cl	CN		8	80	82–84	80–82^63^
7	NO_2_	CN		6	84	158–160	156–158^63^
8	OH	CN		8	73	183–185	180–182^64^
9	H	CO_2_Et		8	67	51–52	49–51^63^
10	OCH_3_	CO_2_Et		7	65	78–80	78–80^63^

Reaction conditions: benzyl alcohol (1 mmol), malononitrile (1 mmol), EtOH (6 mL), catalyst (1.5 mol%), air bubbling, sunlight irradiation, ~ 30 ºC. Structure of the products were identified by NMR spectroscopy (see supporting information). TON (mol of the product per mol of the catalyst = 43.3–58.6), TOF (TON/time) = 5.6–11.7 ^–^h.

**Figure 16:**
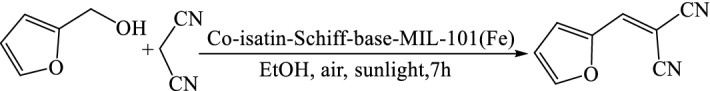
Synthesis of (2-furylmethylene) malononitrile from furfuryl alcohol.

Furthermore, we have found that furfuryl alcohol (Fig. 16) was suitable substrate for photo-oxidation/Knoevenagel reaction under the optimal conditions, and produced the desired Knoevenagel condensation product in 75% yield.

To find the role of Co-isatin-Schiff-base-MIL-101(Fe) in the photo-oxidation/Knoevenagel reaction, the model reaction was studied in the presence of cobalt-free precursors of the catalyst [MIL-101(Fe)–NH_2_ and isatin-Schiff-base-MIL-101(Fe)] or Co-isatin-Schiff-base (Table 9, entries 1–4). A significant decrease in the catalytic efficiency of cobalt-free precursors and Co-isatin-Schiff-base was observed compared with Co-isatin-Schiff-base-MIL-101(Fe). The importance of sunlight irradiation and air in the reaction was also investigated. The product was obtained only in a trace amount under dark conditions (Table 9, entry 5) and alcohol remained intact in the presence of N_2_ atmosphere (Table 9, entry 6).

**Table 9:** Effect of different catalytic species or conditions on tandem one-pot photo-oxidation/Knoevenagel.

Entry	Catalyst	Light irradiation	Time (h)	Yield (%)
1	MIL-101(Fe)–NH_2_	+	5	28
2	Isatin-Schiff-base-MIL-101(Fe)	+	5	38
3^a^	Co-isatin-Schiff-base	+	5	trace
4	Co-isatin-Schiff-base-MIL-101(Fe)	+	5	88
5	Co-isatin-Schiff-base-MIL-101(Fe)	_	5	Trace
6^b^	Co-isatin-Schiff-base-MIL-101(Fe)	+	5	0
7^c^	Co-isatin-Schiff-base-MIL-101(Fe)	+	1.5	92
8^c^	Co-isatin-Schiff-base-MIL-101(Fe)	_	1.5	92
9^b,c^	Co-isatin-Schiff-base-MIL-101(Fe)	+	1.5	92

Reaction conditions: benzyl alcohol (1 mmol, except in entries 8-10), malononitrile (1 mmol), EtOH (6 mL), catalyst (7 mg), air bubbling, sunlight irradiation, ~ 30 °C.

^a^Co-isatin-Schiff-base (isatin-Schiff-base was prepared by the reaction of isatin and aniline).

^b^Under N_2_ atmosphere.

^c^Benzaldehyde (1 mmol).

When benzaldehyde was used instead of benzyl alcohol as the starting material, the target.product was produced more rapidly (Table 9, Entries 7–9). These experiments clearly indicate that the reaction using Co-isatin-Schiff-base-MIL-101(Fe) actually takes place in two key steps: step (1) photo-oxidation of benzyl alcohol to benzaldehyde and step (2) Knoevenagel condensation reaction between the in *situ* formed benzaldehyde and malononitrile. Additional control experiments reveal that Co-isatin-Schiff-base-MIL-101(Fe) act as a photosensitizer which contains Fe (III) and Co (II) counterparts as typical Lewis acidic sites and worked well synergistically.

**Figure 17:**
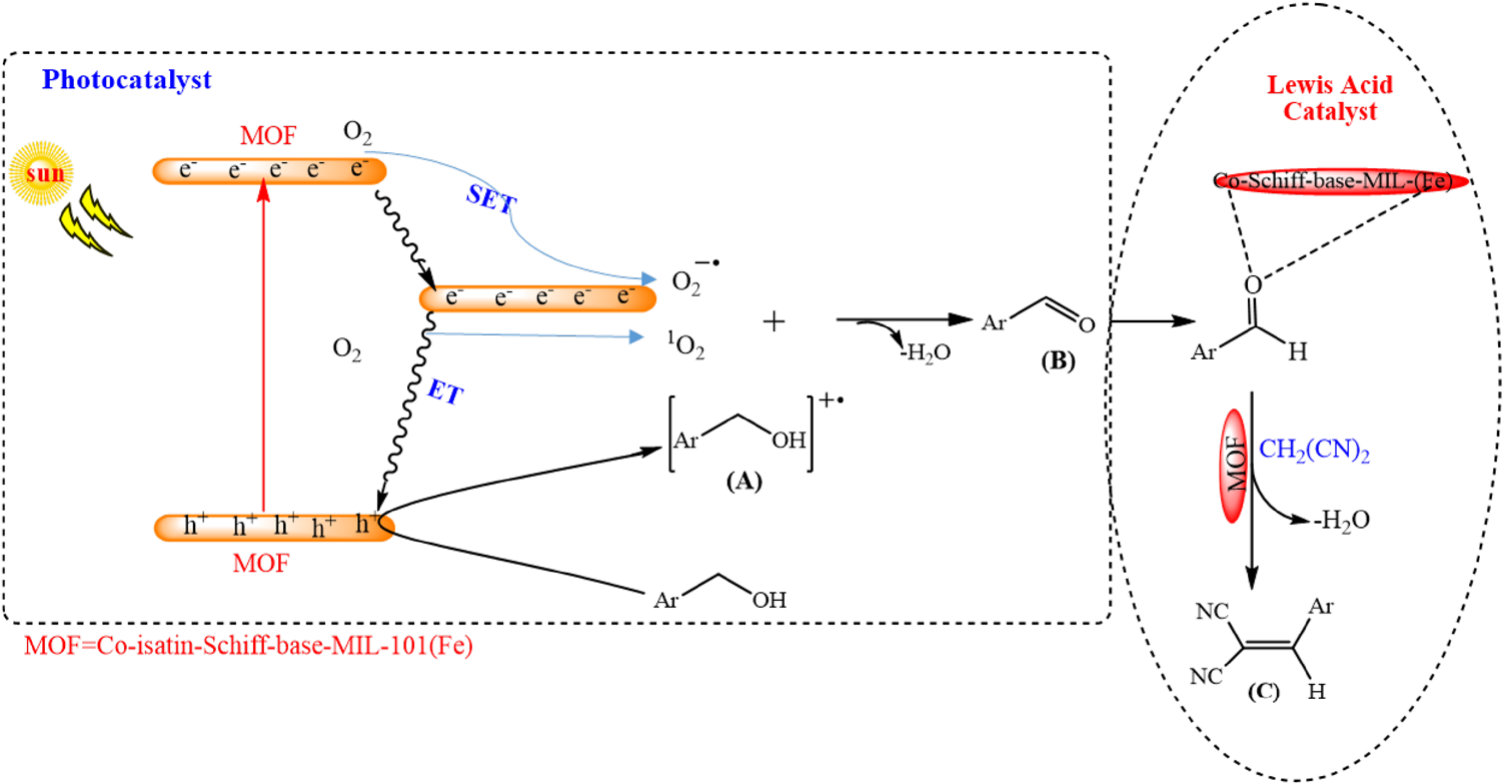
Proposed mechanism for the TOP photo-oxidation/Knoevenagel condensation reaction of alcohols catalyzed by Co-isatin-Schiff-base-MIL-101(Fe).

Hence, according to the control experiments, our observation above and literature survey^32–34^, a possible mechanism for the TOP photo-oxidation/Knoevenagel condensation reaction can be proposed (Fig. 17). The generated hole (h^+^) rapidly oxidizes the benzyl alcohol to create radical cation species (A) as the intermediates, which can consequently react with ROS (O_2_^⋅−^ and ^1^O_2_) to produce the corresponding aldehyde (B). Next, Co-isatin-Schiff-base-MIL-101(Fe) including the Lewis acidic sites (Fe^3+^ and Co^2+^) activates the in situ-produced aldehyde and malononitrile for a nucleophilic addition via Knoevenagel coupling reaction toward the formation of the final product (C).

Finally, the photocatalytic efficiency of the present catalyst was compared with the MOF-based photocatalysts in the benzimidazole synthesis and Knoevenagel condensation starting from alcohols via TOP reported in the literature (Table 10). As represented in Table 10, Co-isatin-Schiff-base-MIL-101(Fe) exhibits the most effective photocatalytic activity for both benzimidazole synthesis and Knoevenagel condensation under sunlight irradiation. The reported methods suffer from drawbacks such as using toxic solvents, high temperature, requiring UV irradiation, prolong reaction time, high catalyst loading and expensive metals. It is worth to mention that because of the high energy of UV photons, which causes the chemical degradation or side reactions, there is a limitation in using UV light irradiation.

**Table 10:** Comparison of photocatalytic efficiency of Co-isatin-Schiff-base-MIL-101(Fe) with some reported MOFs-based photocatalysts for benzimidazole and benzylidenemalononitrile synthesis via one-pot TOP started from alcohols.

Entry^ref^	Catalyst	Product	Reaction conditions	Time (h)	Isolated yield (%)
1^30^	W–ZnO@NH_2_–CBB^a^ (20 mg)	Benzimidazole	EtOH, air, UV–Vis	2	88–99
2^28^	Au/MIL-101 (Fe) (100 mg)	Benzimidazole	EtOH, O_2_, visible light	12	49.5–95.1
3^29^	MIL-100 (Fe) (100 mg)	Benzimidazole	CH_3_CN, O_2_, visible light (300 W Xe)	10	38–96
4^31^	Pt-MIL-101(Fe) (20 mg)	Benzimidazole	CH_3_CN, N_2_, visible light (300 W Xe)	24	63.6–87
5^this work^	Co-isatin-Schiff-base-MIL-101(Fe) (5.3 mg)	Benzimidazole	EtOH, air, sunlight irradiation (∼30 °C)	8	73–90
6^32^	Zr-MOF-NH_2_ (1000 mg)	Benzylidenemalononitrile	*p*-xylene, 90 °C, UV-light irradiation	48	91
7^33^	MIL-101 (Fe)–NH_2_ (200 mg)	Benzylidenemalononitrile	C_6_H_5_CF_3_/CH_3_CN, O_2_, visible light (300 W Xe)	40	20–76
8^34^	PorphCat-Fe^b^ (40 mg)	Benzylidenemalononitrile	CH_3_CN, visible light (∼ 34 °C), O_2_ (1 atm)	24	91
9^This work^	Co-isatin-Schiff-base-MIL-101(Fe) (7 mg)	Benzylidenemalononitrile	EtOH, air, sunlight irradiation (∼ 30 °C)	4–8	65–88

^a^CBB = Coomassie brilliant blue, ^b^porphyrin catecholate iron-based MOF.

### Photocatalytic antibacterial efficiency of Co-isatin-Schiff-base-MIL-101(Fe)

Bacterial pollution intimidates the nutrition, water and community health. Antibiotics are the most famous antibacterial agents for medical treatment of infections caused by bacteria^65,66^. Using a great amount of antibiotics makes bacteria to be resistant towards medicines and so would be lethal for the patients^67,68^. Therefore, introduction of novel and efficient antibacterial agents, which are different from antibiotics is demanded^69^. Nowadays, due to the harmless, efficient, and extensive disinfection influence of semiconductors, they are widely used as antibacterial agents in photocatalytic process. During this process, in the semiconductors, electrons are excited by visible light irradiation and some holes are generated. Then reactive oxygen radicals (HO^⋅^, O_2_^⋅−^) are formed by the reaction of electrons and holes with water and oxygen, which destruct cell walls of bacteria and thus disable the microorganisms^70,71^. MOFs as semiconductors are known as a new generation of antibacterial agents. They have enormous surface area, highly-distributed active sites, tunable porous size, and high biocompatibility and biodegradability^72,73^.

Herein, the photocatalytic antibacterial activity of Co-isatin-Schiff-base-MIL-101(Fe) against *E. coli, S. aureus* and *S. pyogenes* was studied under sunlight illumination. The results showed that almost all bacteria were inactivated after 2 h irradiation in the presence of MOF as a bio-photo-antibacterial agent, with the antibacterial efficiency of 99.57%, 99.85% and 99.48%, for *E. coli, S. aureus* and *S. pyogenes*, respectively. The negligible decreasing in the bacterial density treated with photocatalyst in the dark or without Co-isatin-Schiff-base-MIL-101(Fe) at light conditions indicates that both light irradiation and photocatalyst are necessary for the efficient antibacterial activity. Moreover, minimal inhibitory concentration (MIC) and minimal bactericidal concentration (MBC) values for *E. coli* and *S. aureus* are the same and are higher in the case of *S. pyogenes* (Table 11).

**Table 11.** The antibacterial test results of Co-isatin-Schiff-base-MIL-101(Fe) for different bacterial strains: *E. coli*, *S. aureus* and *S. pyogenes* under sunlight irradiation.

Bactria	MIC^a^	MBC^a^
*E. coli*	250	500
*S. aureus*	250	500
*S. pyogenes*	500	1000

^a^Minimal inhibitory concentration (MIC) and minimal bactericidal concentration (MBC) values were reported as μg mL^−1^.

## Conclusion

In conclusion, a metal–organic framework functionalized with cobalt-complex [Co-isatin-Schiff-base-MIL-101(Fe)] as a heterogeneous multifunctional bio-photocatalyst for the synthesis of benz-imidazoles/-oxazoles/-thiazoles, and benzylidene malononitrile by tandem oxidation process starting from readily available alcohols is introduced. In this method, photo-oxidation of alcohols and cyclocondensation of the *in-situ* formed aldehydes with *o*-substituted aniline (*o*-phenylenediamine/*o*-aminophenol/*o*-aminothiophenol) or malononitrile affords benz-imidazoles/-oxazoles/-thiazoles, or benzylidene malononitrile, respectively, in one-pot operation in good to high yields. Co-isatin-Schiff-base-MIL-101(Fe) acts both as a bio-photocatalyst to produce reactive ^1^O_2_, O_2_^⋅−^, and a Lewis acid to catalyze the reaction of the *in-situ* formed aldehydes with *o*-substituted anilines or malononitrile. A significant decrease in the band gap energy and an increase in the characteristic emission of MIL-101(Fe) after functionalization with cobalt Schiff-base according to DRS analysis and fluorescence spectroscopy, respectively, indicate that the photocatalytic efficiency of Co-isatin-Schiff-base-MIL-101(Fe) is related primarily to the synergetic effect of Fe–O cluster and Co-Schiff-base. EPR results obviously point out that Co-isatin-Schiff-base-MIL-101(Fe) is capable of generating ^1^O_2_ and O_2_^⋅−^ as active oxygen species under visible light irradiation. The use of sunlight and air as abundant and cheap sources without needing any specific chemical oxidizing agent, using low loading of a reusable bio-photocatalyst in ethanol as an environmentally friendly solvent, and easy separation of the obtained products are the advantages of this method. Moreover, Co-isatin-Schiff-base-MIL-101(Fe) exhibits outstanding sunlight-induced photocatalytic antibacterial activity for the inactivation of three kinds of bacteria. Based on our knowledge, this is the first report of using a bio-photocatalyst for the synthesis of the target molecules.

## Supplementary Information

Supplementary Information.

## Data Availability

All data generated or analysed during this study are included in this published article (and its Supplementary Information files).
