# Supplementary material for: Cobalt isatin-Schiff-base derivative of MOF as a heterogeneous multifunctional bio-photocatalyst for sunlight-induced tandem air oxidation condensation process
Source: Sci Rep. 2023 Mar 29;13:5115. doi: 10.1038/s41598-023-32241-z (PMC10060412; doi:10.1038/s41598-023-32241-z)
Supplement: Supplementary file 1 — Supplementary Information. [file 41598_2023_32241_MOESM1_ESM.docx]

Cobalt isatin-Schiff-base derivative of MOF as a heterogeneous multifunctional bio-photocatalyst for sunlight-induced tandem air oxidation condensation process

*Majid Rouzifar,^a^ Sara Sobhani,^*a^ Alireza Farrokhi^a^ and José Miguel Sansano^b^*

Address: Department of Chemistry, College of Sciences, University of Birjand, Birjand, Iran, email: [ssobhani@birjand.ac.ir](mailto:ssobhani@birjand.ac.ir)

Departamento de Qu´ımica Org´anica, Facultad de Ciencias, Centro de Innovaci´on en Qu´ımica Avanzada (ORFEO-CINQA) and Instituto de S´ıntesis Org´anica (ISO), Universidad de Alicante, Apdo. 99, 03080-Alicante, Spain

**Photocatalytic antibacterial experiments**

Co-isatin-Schiff base MIL-101(Fe) (50 μL) was mixed with 150 μL of bacteria (at approximately 1.5 x 10 7 CFU/mL^-1^) in a 96-well plate. All samples were tested under two conditions: under sunlight irradiation or dark conditions for 2 h. Next, 15 μL of each sample was added to 3 mL of culture medium. 30 μL of which was plated on an agar plate and kept at 37°C for 24 hours to observe colony numbers. A bacterial solution mixed with 50 μL of ultrapure water was used as a control group.

**General information**

X-ray photoelectron spectroscopy (XPS) was performed by a VG-Microtech Multilab 3000 spectrometer, equipped with an Al anode. The deconvolution of spectra was done by Gaussian Lorentzian curves. The ^1^HNMR spectra were recorded in a Brucker Advance 300 MHz spectrometers using CDCl_3_ and DMSO-d_6_ and solvents containing tetramethylsilane as the internal standard.


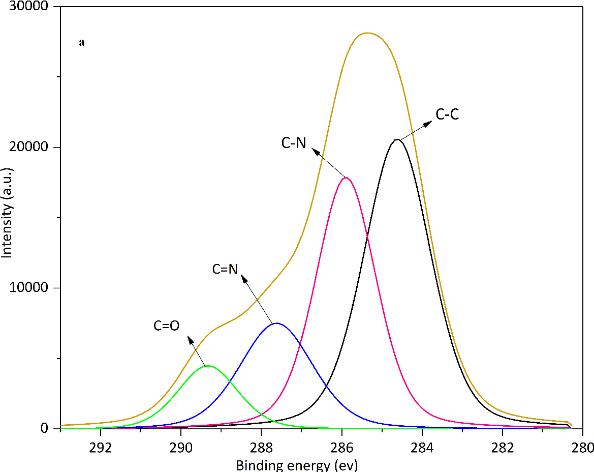

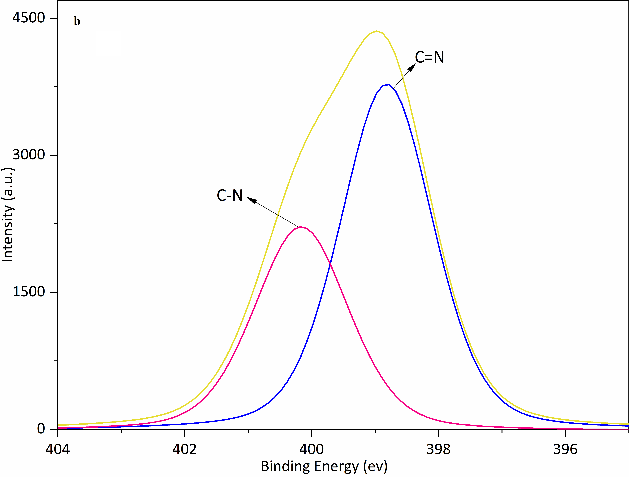

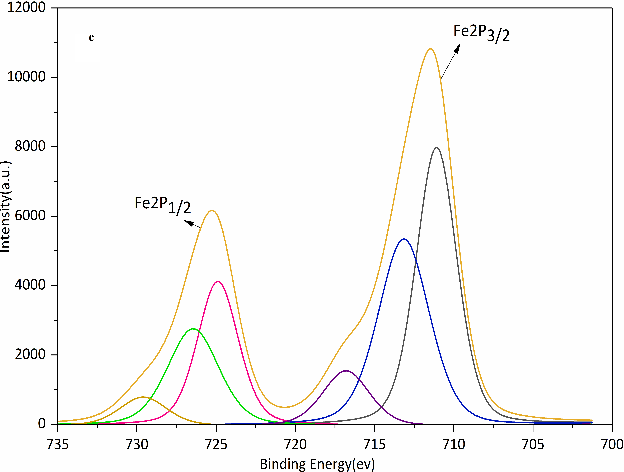

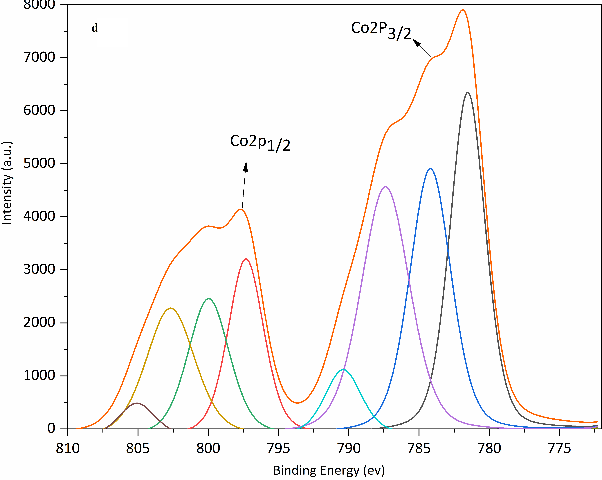


**Figure S1**. (a) XPS fingerprints of the Co-isatin-Schiff-base-MIL-101(Fe), (a) C 1s, (b) N 1s, (c) Fe 2p and (d) Co.


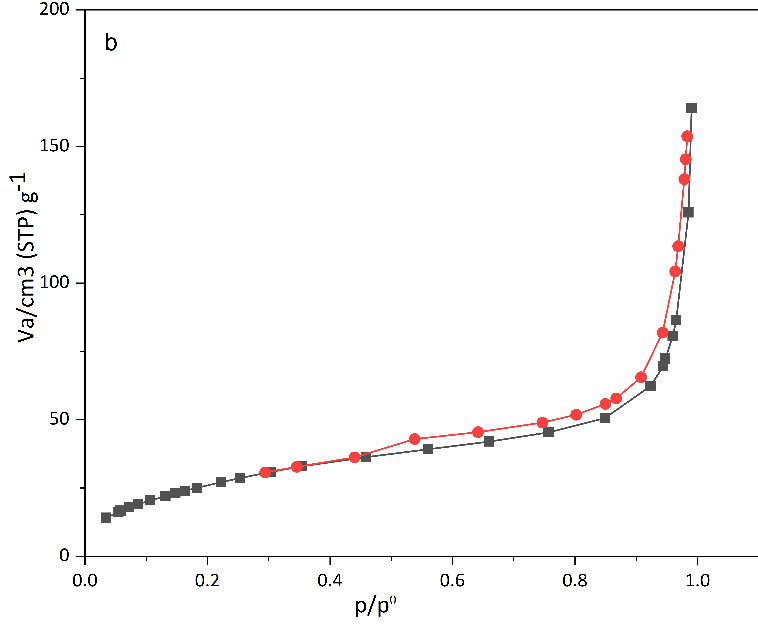

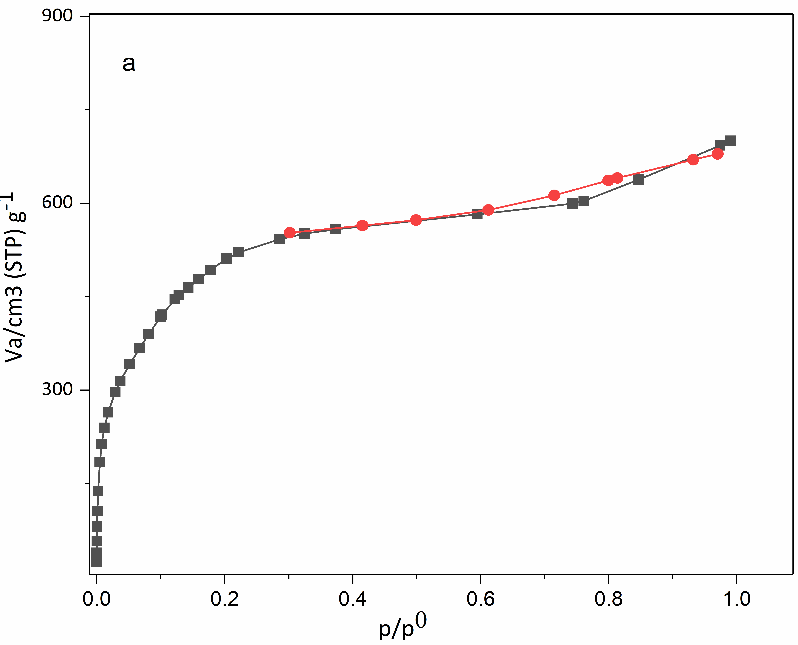


C

**Figure S2**. N_2_ adsorption-desorption of (a) Fe-MIL-101-NH_2_ and (b) Fe-MIL-101-isatin-Schiffbase-Co, pore size distribution of (c) Fe-MIL-101-NH_2_ and (d) Fe-MIL-101-isatin-Schiffbase-Co.


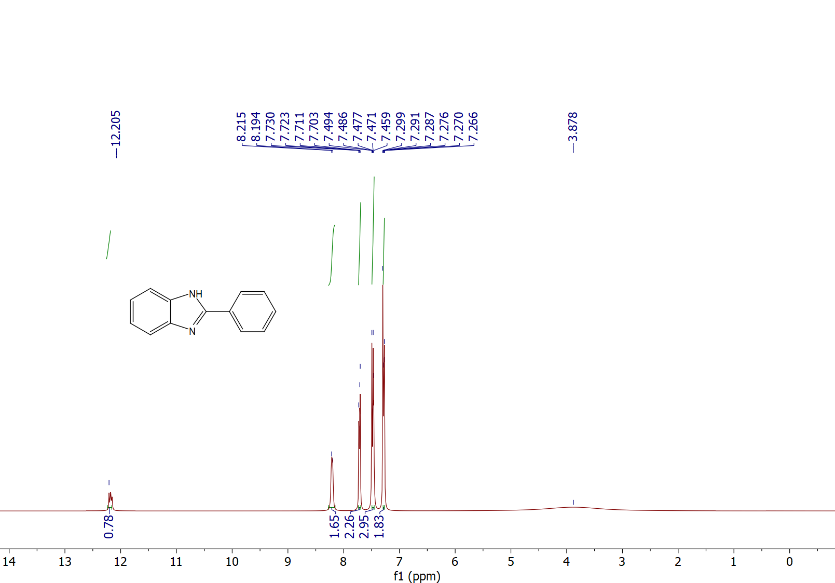


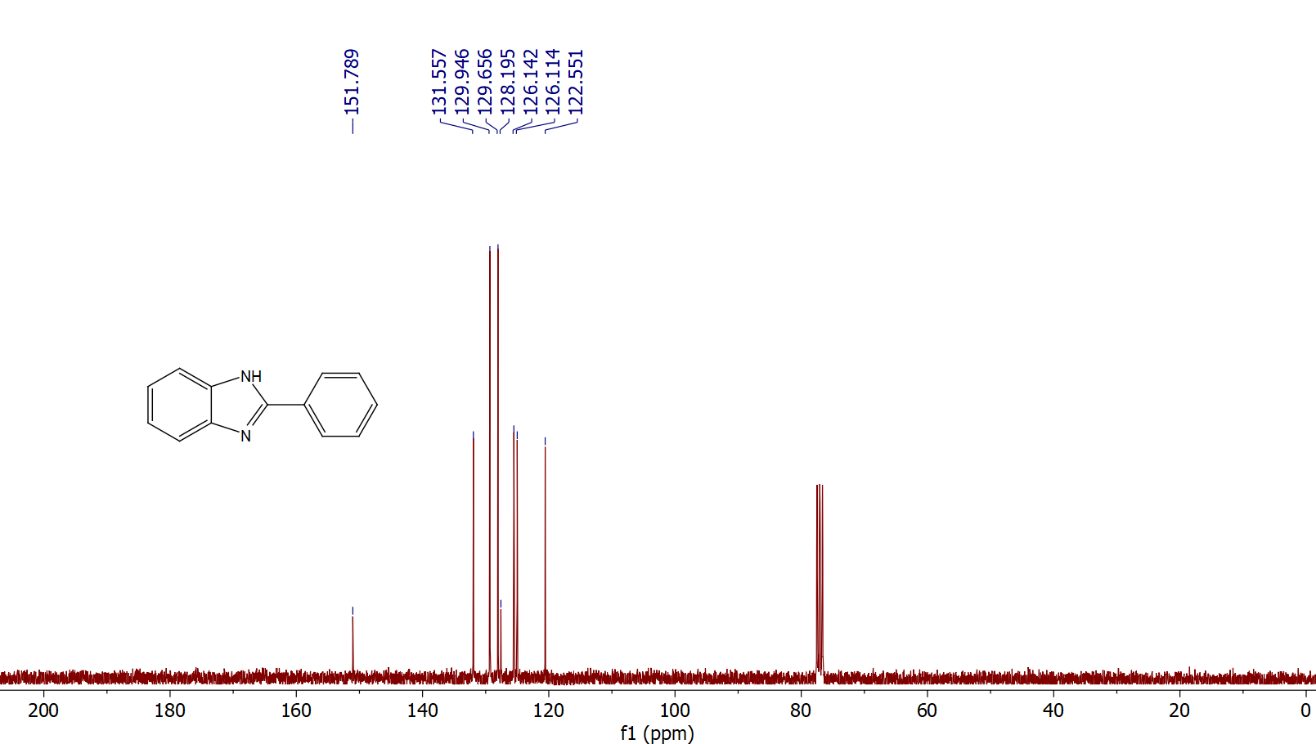


**Figure S3*.*** ^1^H NMR and ^13^C NMR spectra of 2-phenyl-benzoimidazole

^1^HNMR (300 MHz, CDCl_3_): δ 12.20 (s, 1H), 8.20 (d, *J* = 6.3 Hz, 2H), 7.73 –7.70 (m, 2H), 7.49-7.46 (m, 3H), 7.30-7.27 (m, 2H) ppm.^13^CNMR (76 MHz, CDCl_3_): δ 151.8, 131.6, 129.9, 129.7, 128.2, 126.2, 126.1, 122.6 ppm.


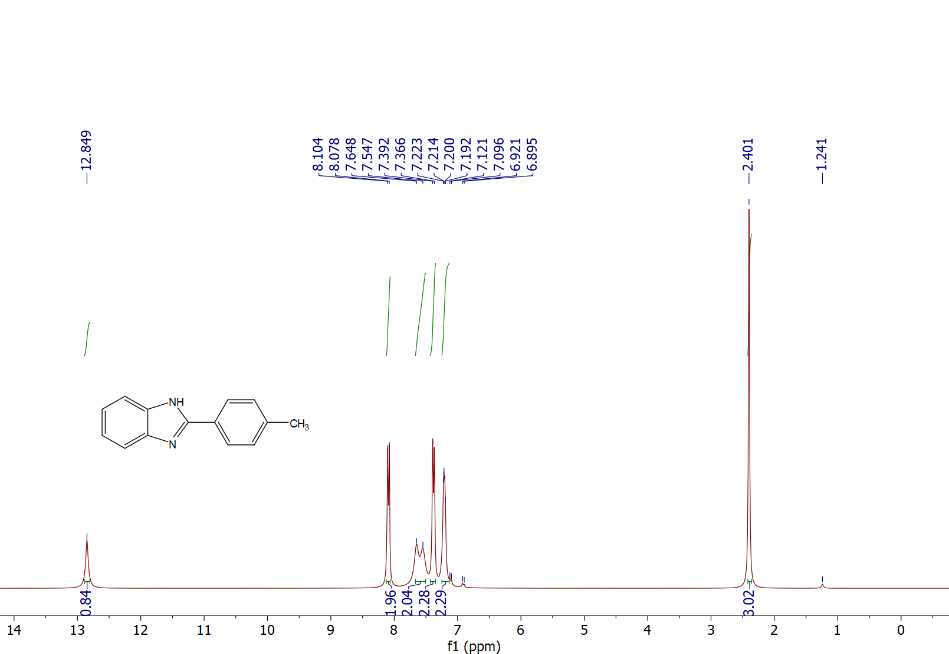


**
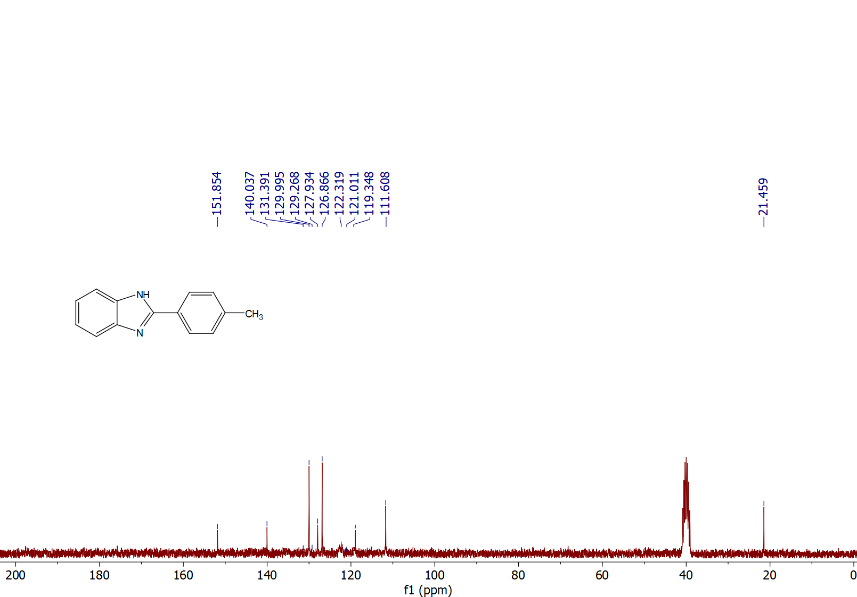
**

**Figure S4*.*** ^1^H NMR and ^13^C NMR spectra of 2-(4-methylphenyl)-benzoimidazole

^1^HNMR (300 MHz, DMSO): δ 12.85 (s, 1H), 8.09 (d, *J* = 7.8 Hz, 2H), 7.65 –7.54 (m, 2H), 7.38 (d, *J* = 7.8 Hz, 2H), 7.21 (dd, *J* = 6.6 Hz, *J* = 2.5 Hz, 2H), 2.40 (s, 3H) ppm.^13^CNMR (76 MHz, DMSO): δ 151.9, 140.0, 131.4, 130.0, 129.3, 127.9,126.9, 122.3, 121.0, 119.3, 111.6, 21.5 ppm.


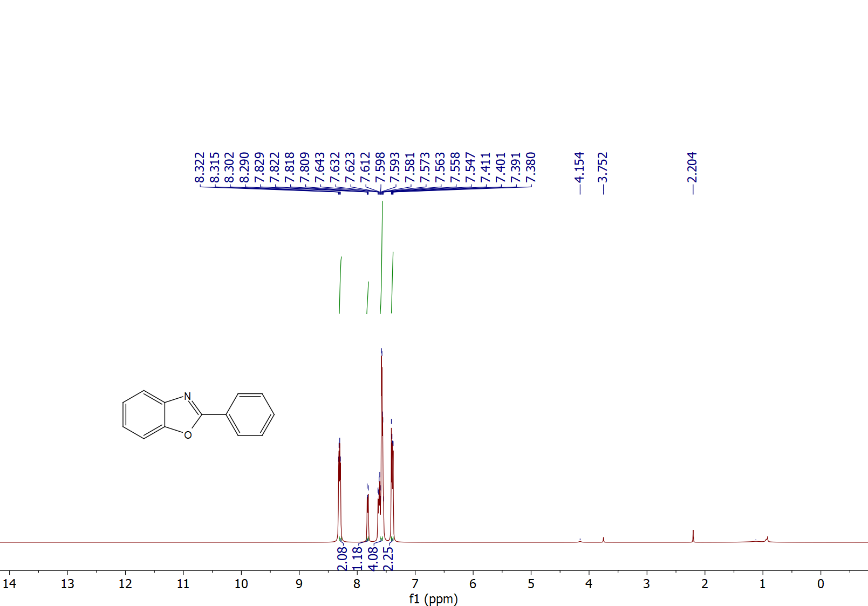


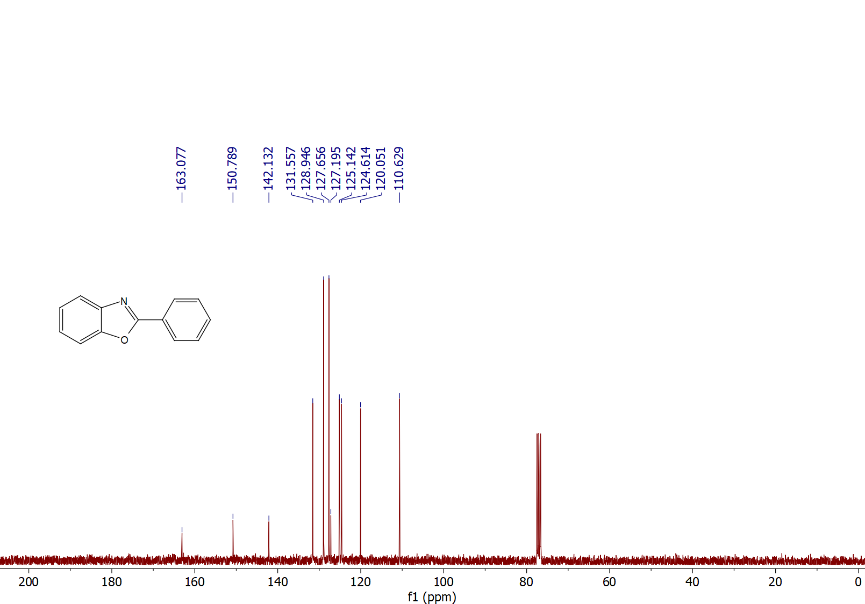


**Figure S5*.*** ^1^H NMR and ^13^C NMR spectra of 2-phenylbenzoxazole

^1^HNMR (300 MHz, CDCl_3_): δ 8.32-8.29 (m, 2H), 7.83-7.81 (m, 1H), 7.64-7.55 (m, 4H), 7.341-7.38 (m, 2H) ppm.^13^CNMR (76 MHz, CDCl_3_): δ 163.1, 150.8, 142.1, 131.6, 128.9, 127.7, 127.2, 125.1, 124. 7, 120.1, 110.6 ppm.

**
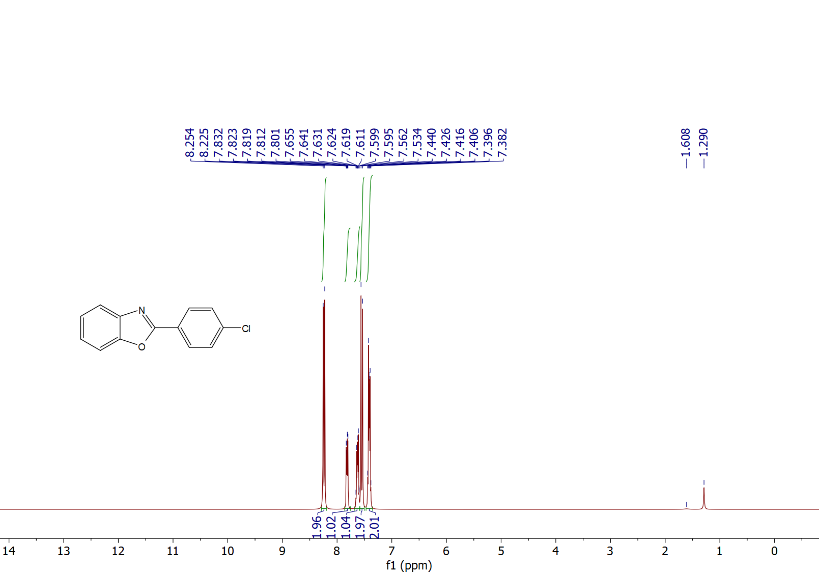
**

**
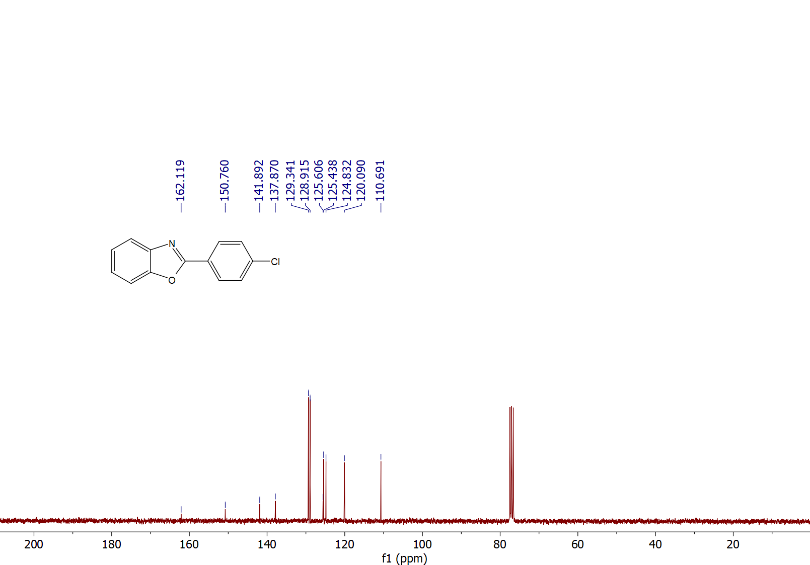
**

**Figure S6***.* ^1^H NMR and ^13^C NMR spectra of 2-(4-chlorophenyl)-benzoxazole

^1^HNMR (CDCl_3_, 300 MHz): δ 8.24 (d, 2H, *J* = 8.7 Hz, 2H), 7.83-7.80 (m, 1H), 7.66-7.60 (m, 1H), 7.54 (d, *J* = 8.4 Hz, 2H), 7.44-7.38 (m, 2H) ppm. ^13^CNMR (76 MHz, CDCl_3_): δ 162.1, 150.8, 141.9, 137.9, 129.3, 128.9, 125.6, 125.4, 124.8, 120.1, 110.7 ppm.

**
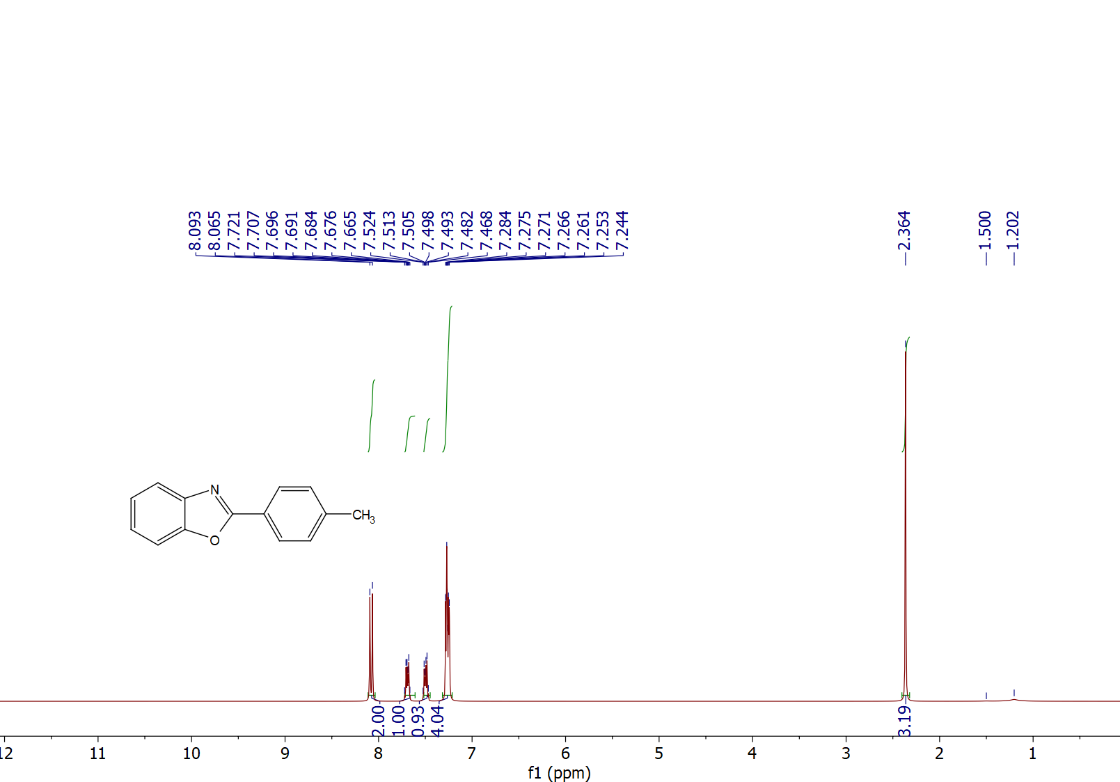
**

**
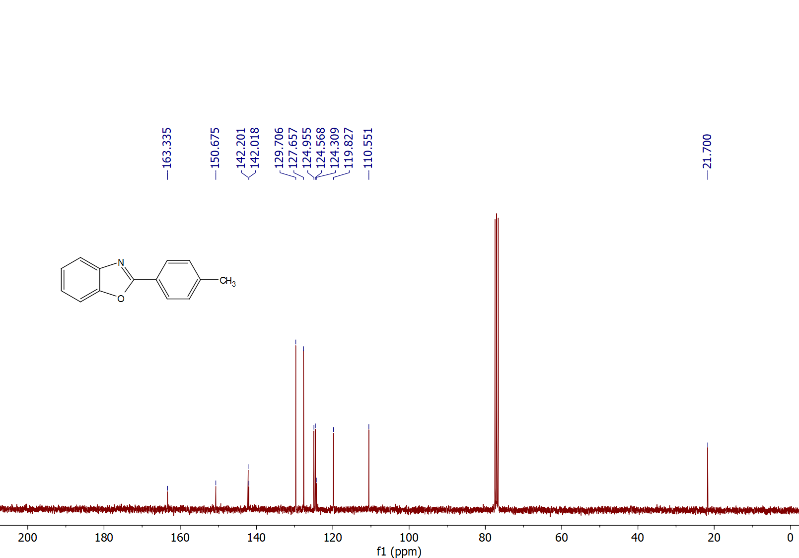
**

**Figure S7*.***^1^H NMR and ^13^C NMR spectra of 2-(4-methyphenyl)-benzoxazole

^1^HNMR (300 MHz, CDCl_3_): δ 8.07 (d, *J* = 8.4 Hz, 2H), 7.72-7.66 (m, 1H), 7.52-7.46 (m, 1H), 7.28-7.24 (m, 4H), 2.36 (s, 3H) ppm. ^13^CNMR (76 MHz, CDCl_3_): δ 163.3, 150.7, 142.2, 142.0, 129.7, 127.7, 125.0, 124.6, 124.3, 119.8, 110.6, 21.7 ppm.


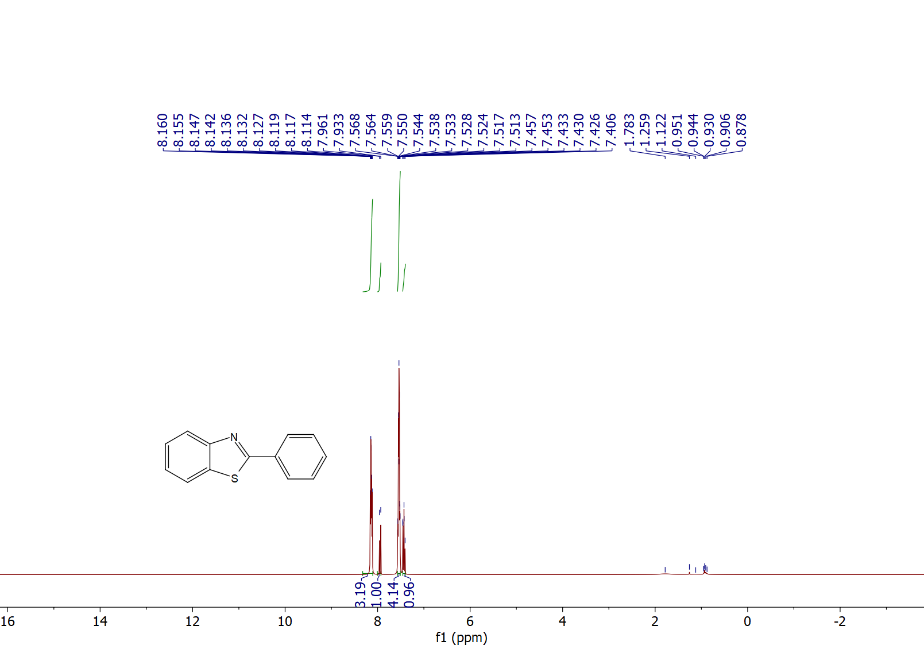


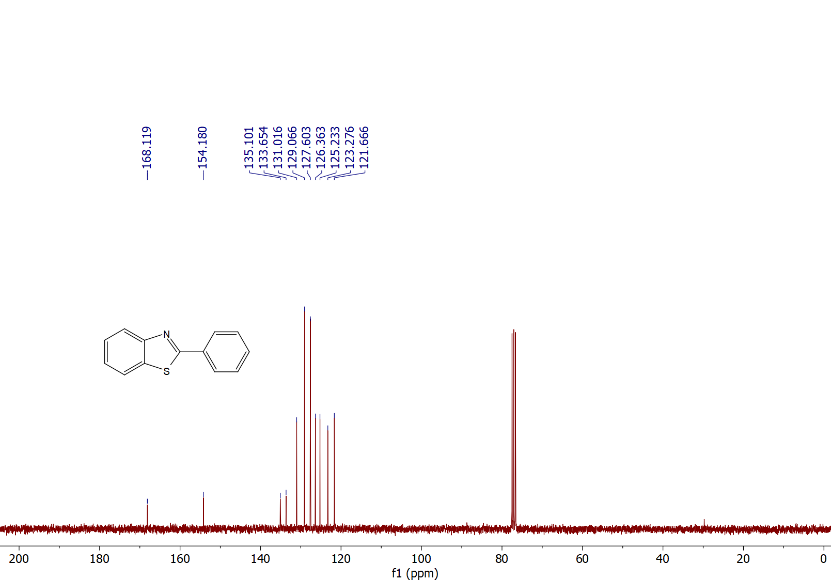


**Figure S8*.*** ^1^H NMR and ^13^C NMR spectra of 2-phenylbenzothiazole

^1^HNMR (CDCl_3_, 300 MHz): δ 8.16-8.11 (m, 3H), 7.95 (d, *J* = 8.4 Hz, 1H), 7.57-7.51 (m, 4H), 7.46-7.41 (m, 1H) ppm.^13^CNMR (76 MHz, CDCl_3_): δ 168.1, 154.2, 135.1, 133.7, 131.0, 129.1, 127.6, 126.4, 125.2, 123.3, 121.7 ppm.


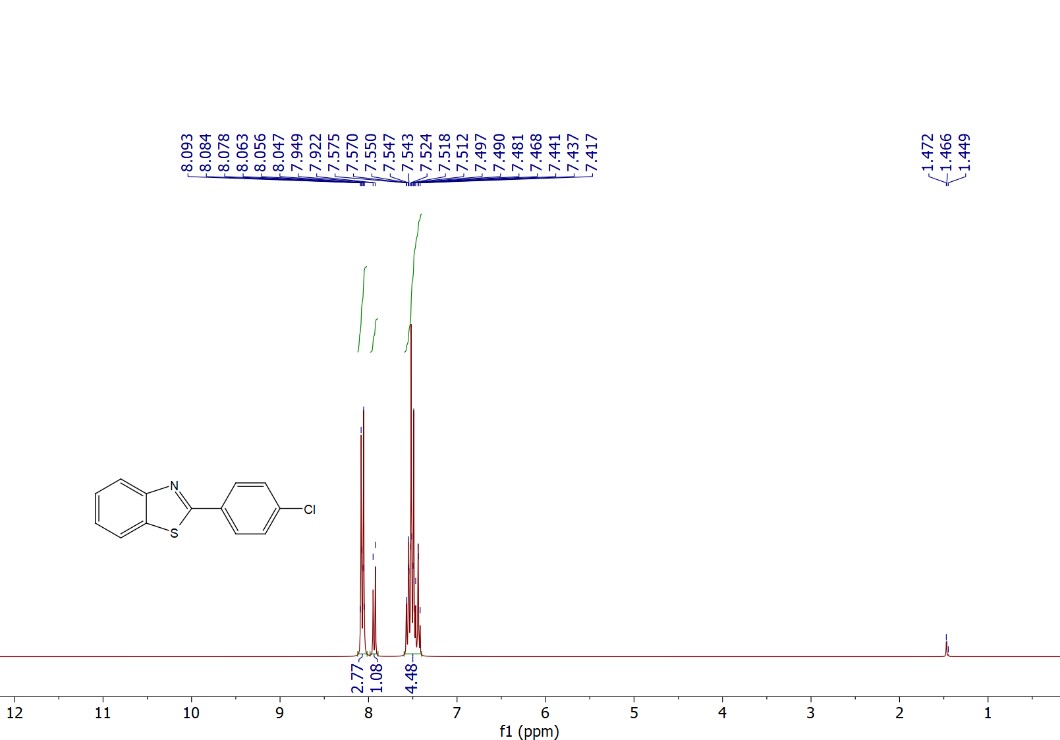
.


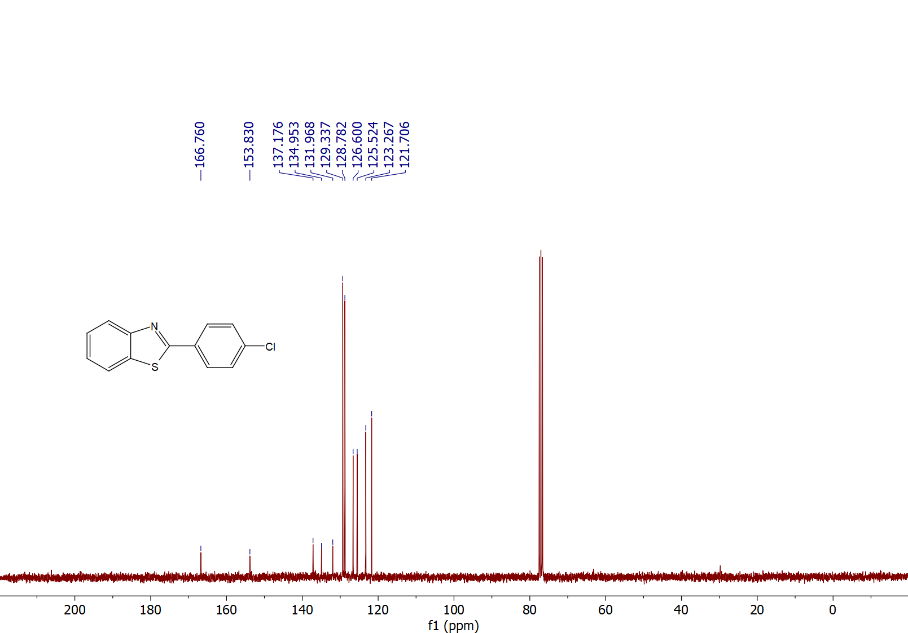


**Figure S9*.*** ^1^H NMR and ^13^C NMR spectra of 2-(4-chlorophenyl)-benzthiazole

^1^HNMR (300 MHz, CDCl_3_): δ 8.09-8.05 (m, 3H), 7.94 (d, *J* = 8.1 Hz, 1H), 7.58-7.42 (m, 4H) ppm. ^13^CNMR (76 MHz, CDCl_3_): δ 166.8, 153.8, 137.2, 135.0, 132.0, 129.3, 128.8, 126.6, 125.5, 123.3, 121.7 ppm.


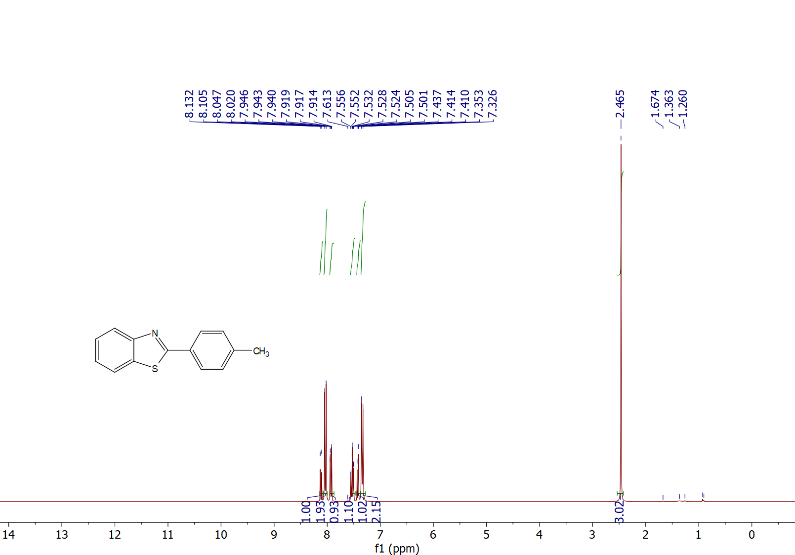

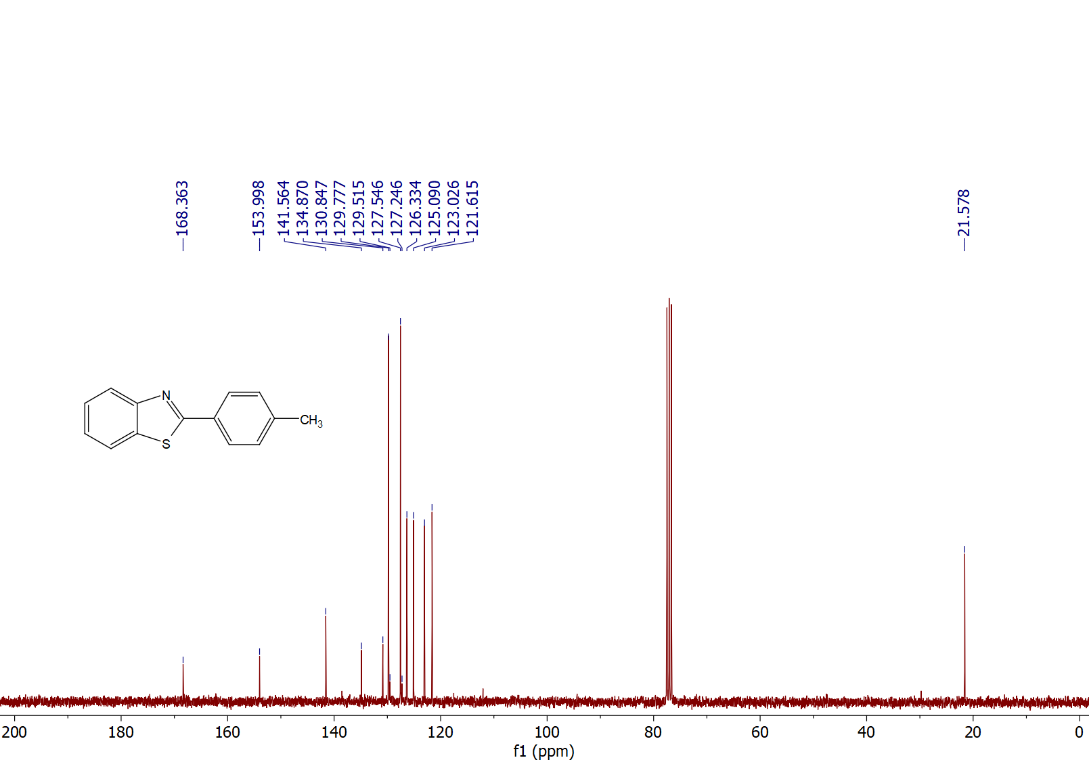


**Figure S10*.*** ^1^H NMR and ^13^C NMR spectra of 2-(4-methylphenyl)-benzthiazole

^1^HNMR (300 MHz, CDCl_3_): δ 8.11 (d, *J* = 8.1 Hz, 1H), 8.03 (d, *J* = 8.1 Hz, 2H), 7.95-7.91 (m, 1H), 7.55-7.50 (m, 1H), 7.43-7.41 (m, 1H), 7.33 (d, *J* = 8.1 Hz, 2H), 2.46 (s, 3H) ppm. ^13^CNMR (76 MHz, CDCl_3_): δ 168.4, 154.0, 141.6, 134.8, 130.8, 129.8, 129.5, 127.5, 127.3, 126.3, 125.1, 123.0, 121.6, 21.6 ppm.


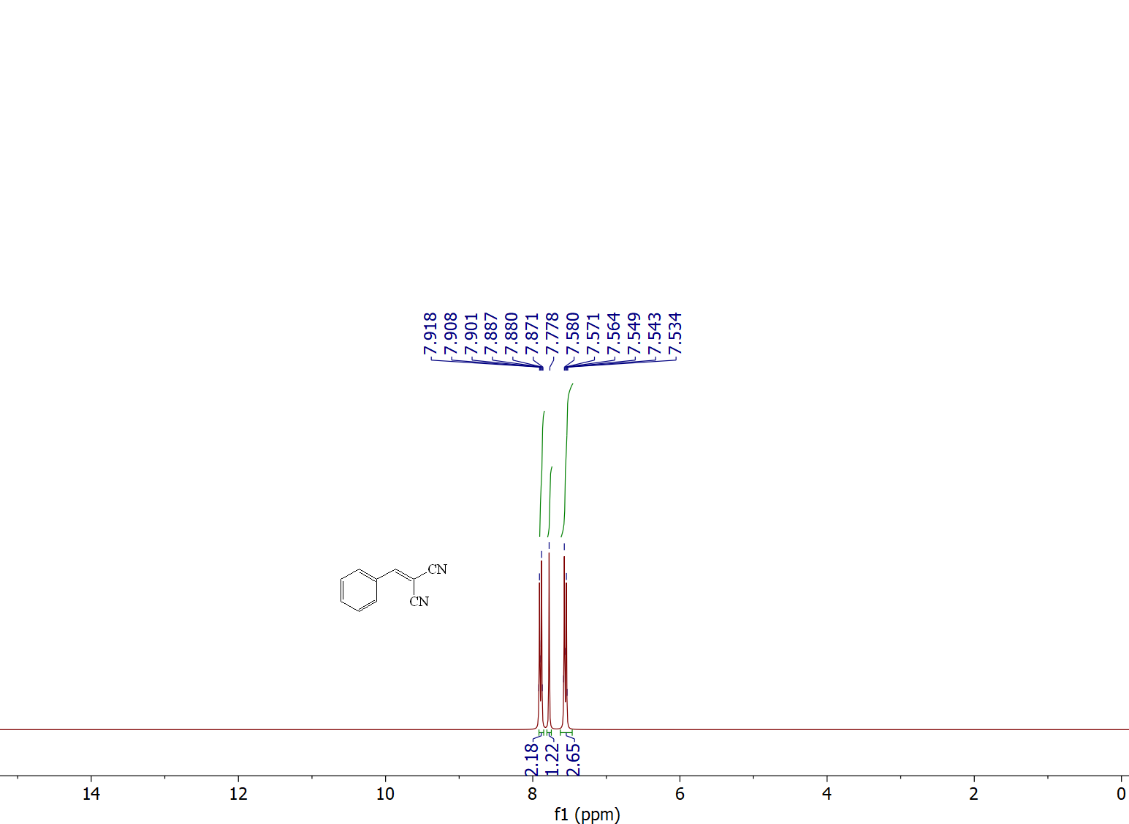


**
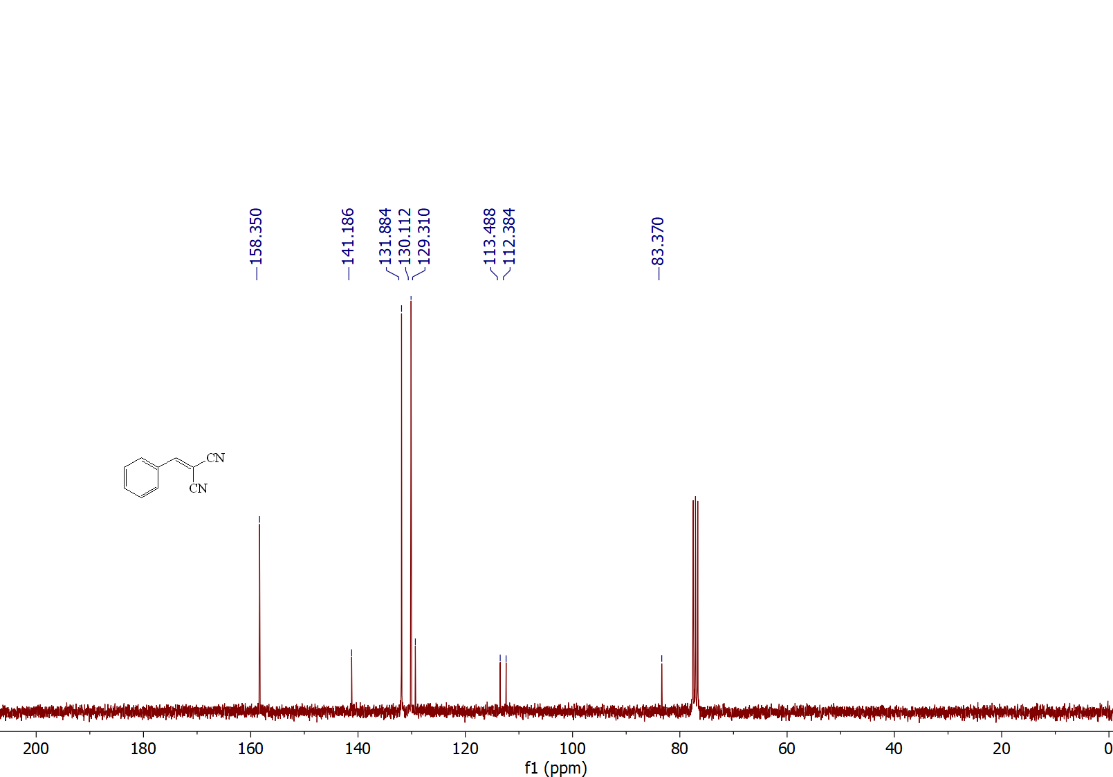
**

**Figure S11*.*** ^1^H NMR and ^13^C NMR spectra of 2-benzylidenemalononitrile

^1^HNMR (300 MHz, CDCl_3_) δ 7.92-7.87 (m, 2H), 7.78 (s, 1H), 7.58-7.53 (m, 3H) ppm. ^13^CNMR (76 MHz, CDCl_3_): δ 158.4, 141.2, 131.9, 130.1, 129.3, 113.5, 112.4, 83.4 ppm.


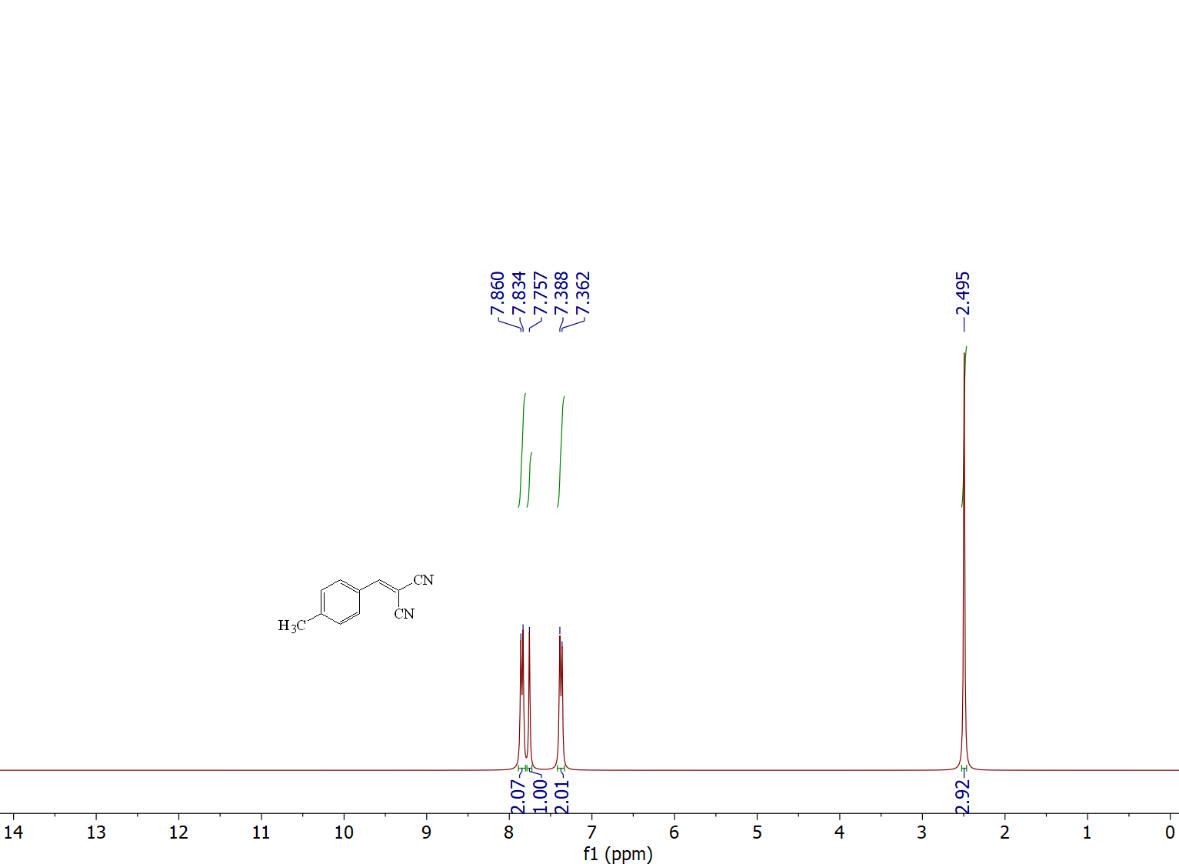


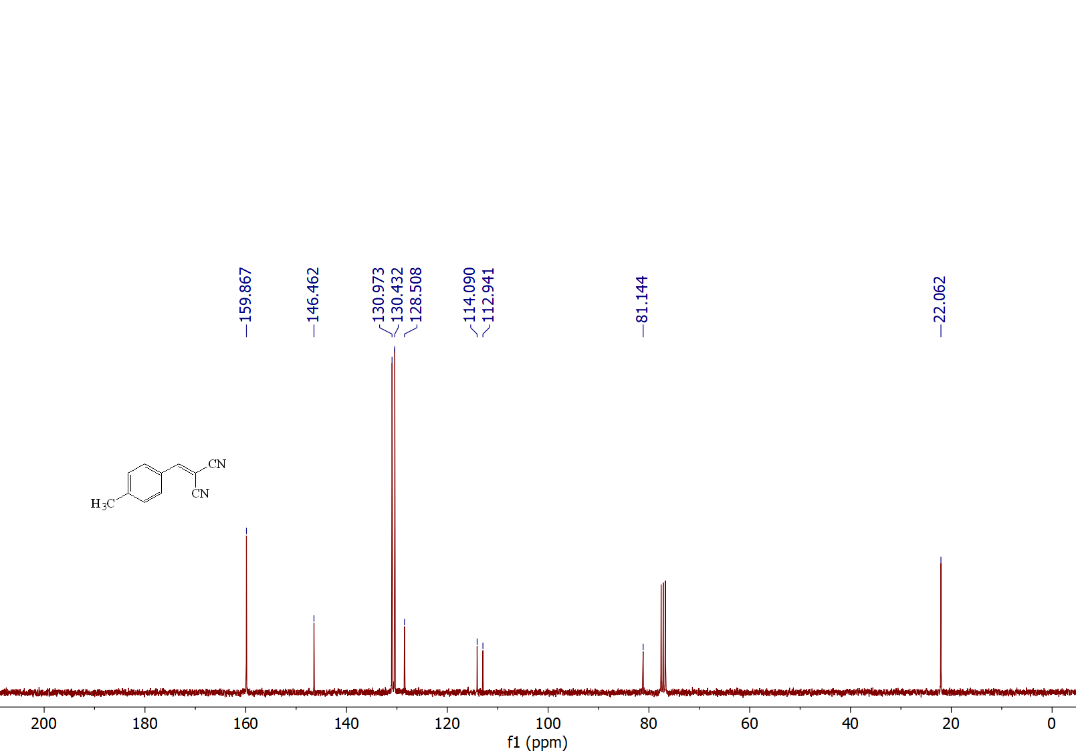


**Figure S12*.*** ^1^H NMR and ^13^C NMR spectra of 2-(4-methylbenzylidene) malononitrile

^1^HNMR (300 MHz CDCl_3_): δ 7.85 (d, *J* = 7.8 Hz, 2H), 7.76 (s, 1H), 7.37 (d, *J* = 7.8 Hz, 2H), 2.49 (s, 3H) ppm. ^13^CNMR (76 MHz, CDCl_3_): δ 159.9, 146.5, 131.0, 130.4, 128.5, 114.1, 112.9, 81.1, 22.1 ppm.

**
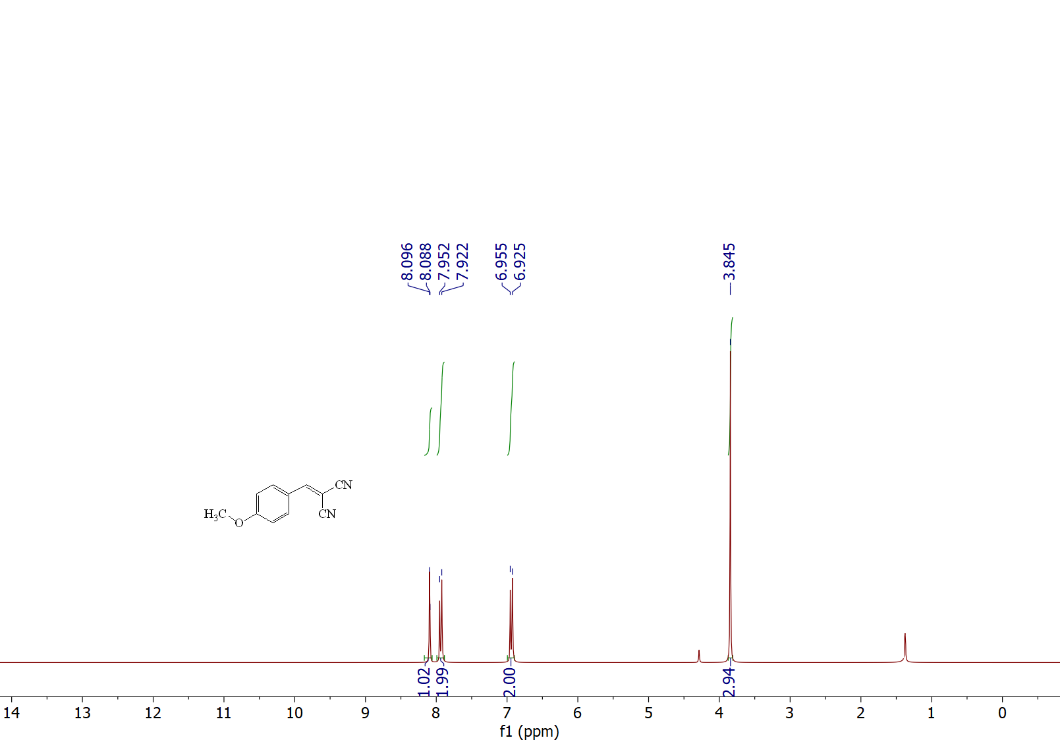
**


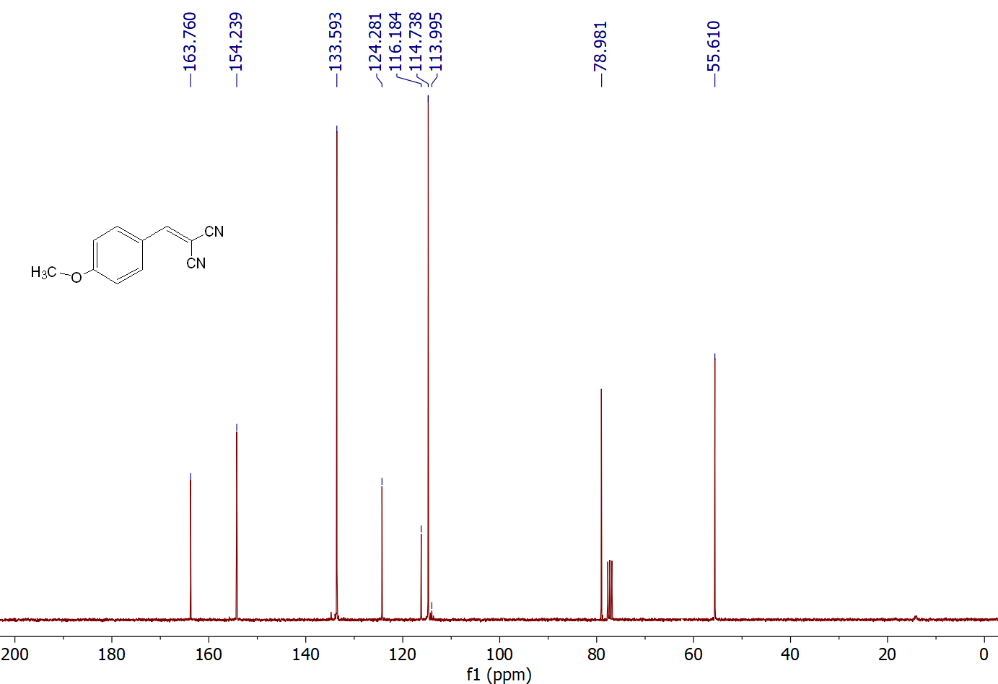


**Figure S13*.*** ^1^H NMR and ^13^C NMR spectra of 2-(4-methoxybenzylidene)malononitrile

^1^HNMR (300MHz, CDCl_3_): δ 8.09-8.08 (m, 1H), 7.96-7.92 (m, 2H), 6.96-6.92 (m, 2H) ppm. ^13^CNMR (76 MHz, CDCl_3_): δ 163.8, 154.2, 133.6, 124.3, 116.2, 114.7, 114.0, 79.0, 55.6 ppm.


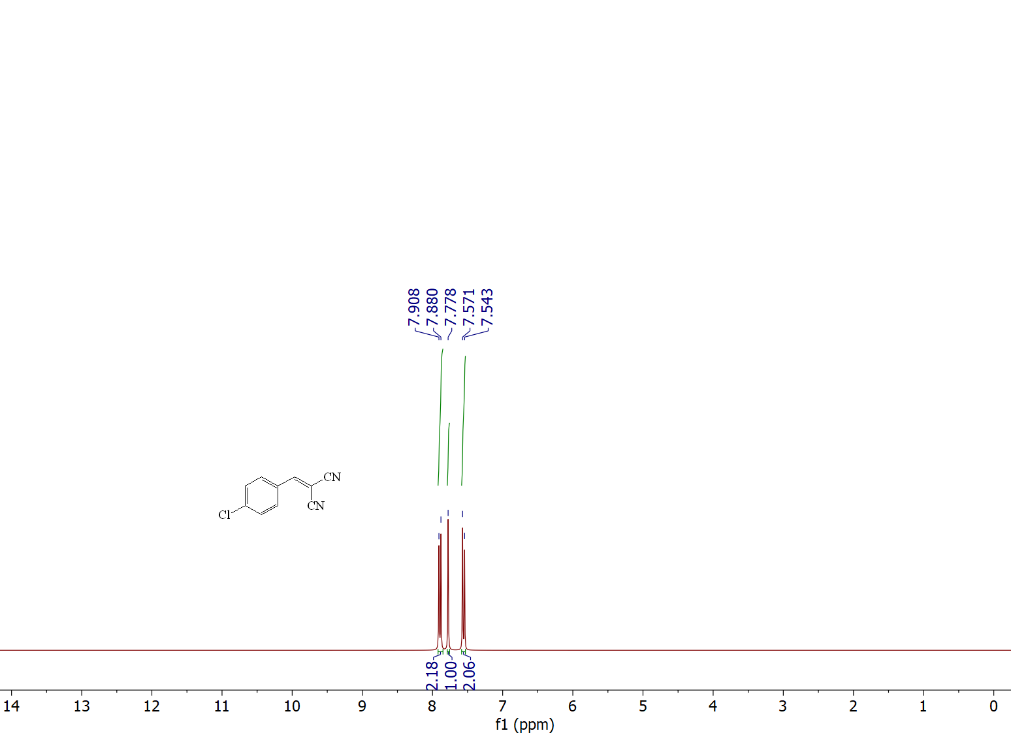


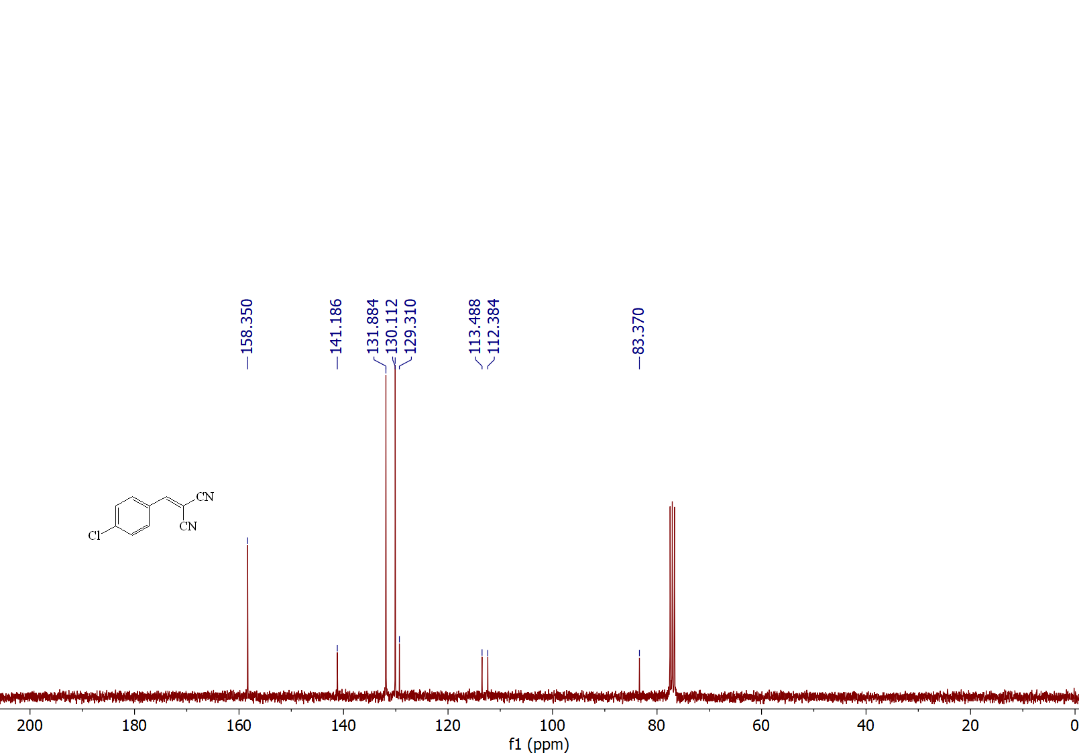


**Figure S14*.*** ^1^H NMR and ^13^C NMR spectra of 2-(4-chlorobenzylidene)malononitrile

^1^HNMR (300 MHz, CDCl_3_): δ 7.89 (d, *J* = 8.4 Hz, 2H), 7.78 (s, 1H), 7.56 (d, *J* = 8.4 Hz, 2H) ppm. ^13^CNMR (76 MHz, CDCl_3_): δ 158.4, 141.2, 131.9, 130.1, 129.3, 113.5, 112.4, 83.4 ppm).


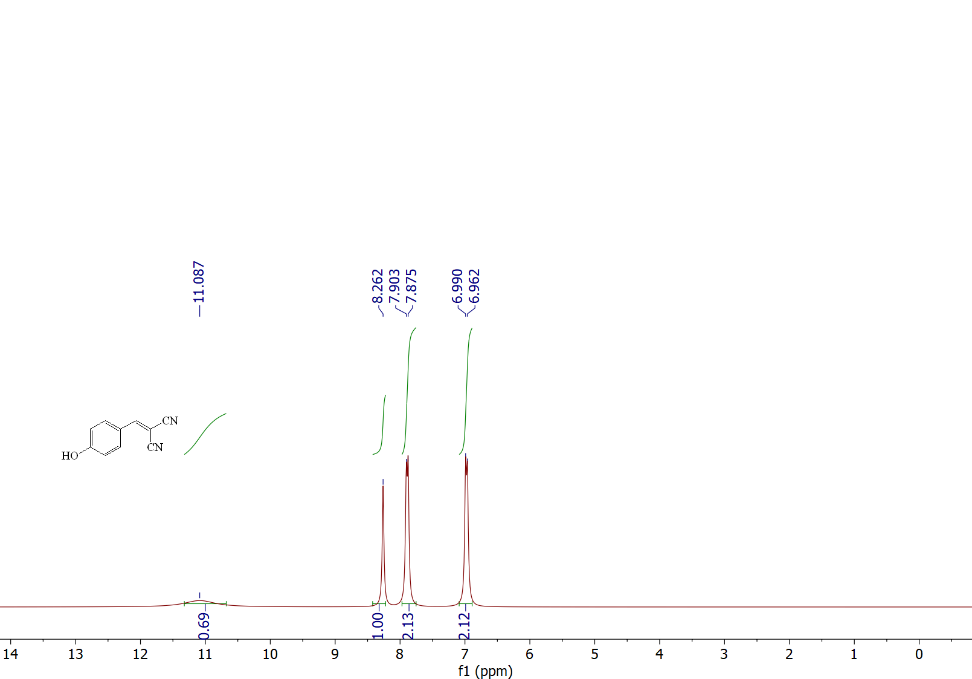


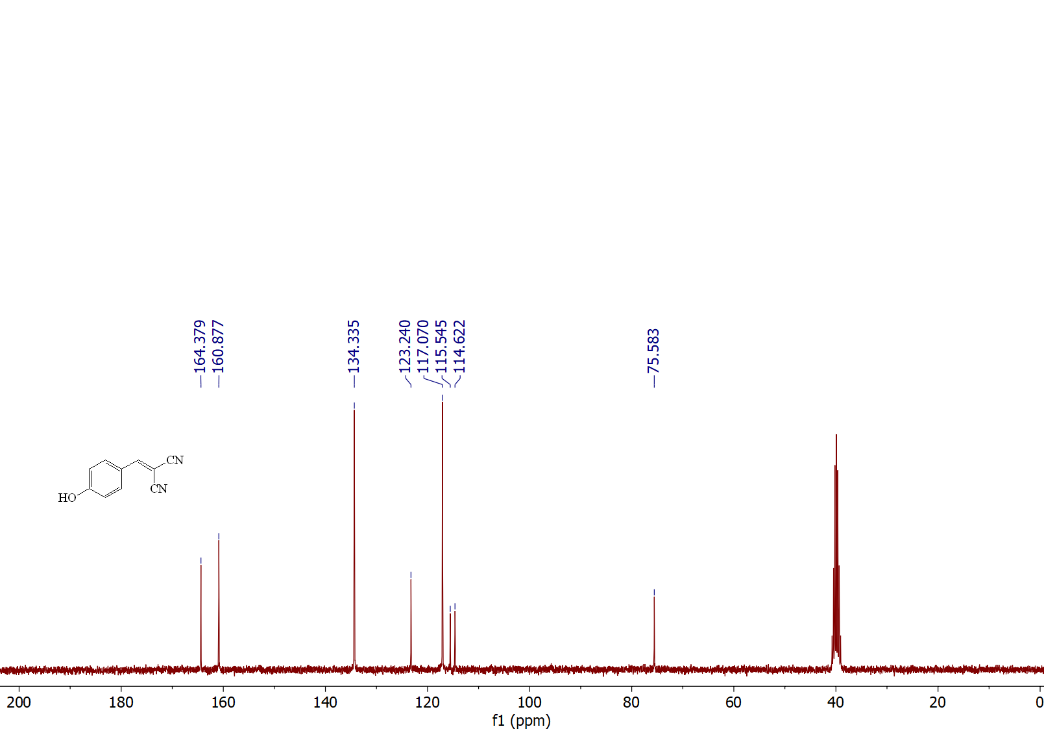


**Figure S15*.*** ^1^H NMR and ^13^C NMR spectra of 2-(4-hydroxybenzylidene) malononitrile

^1^HNMR (300 MHz, CDCl_3_): δ 11.08 (bs, 1H), 8.26 (s, 1H), 7.89 (d, *J* = 8.4 Hz, 2H), 6.98 (d, *J* = 8.4 Hz, 2H) ppm. ^13^CNMR (76 MHz, CDCl_3_): δ 164.4, 160.9, 134.3, 123.2, 117.1, 115.5, 114.6, 75.6 ppm.


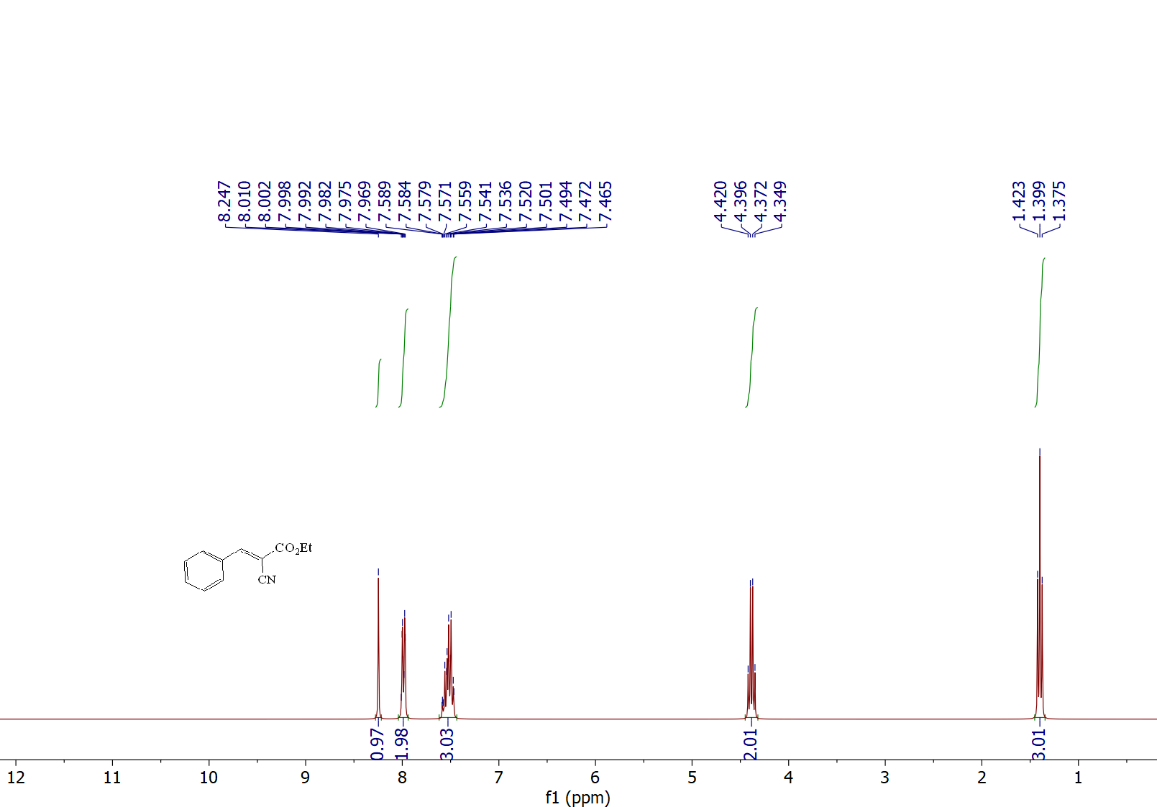


**^
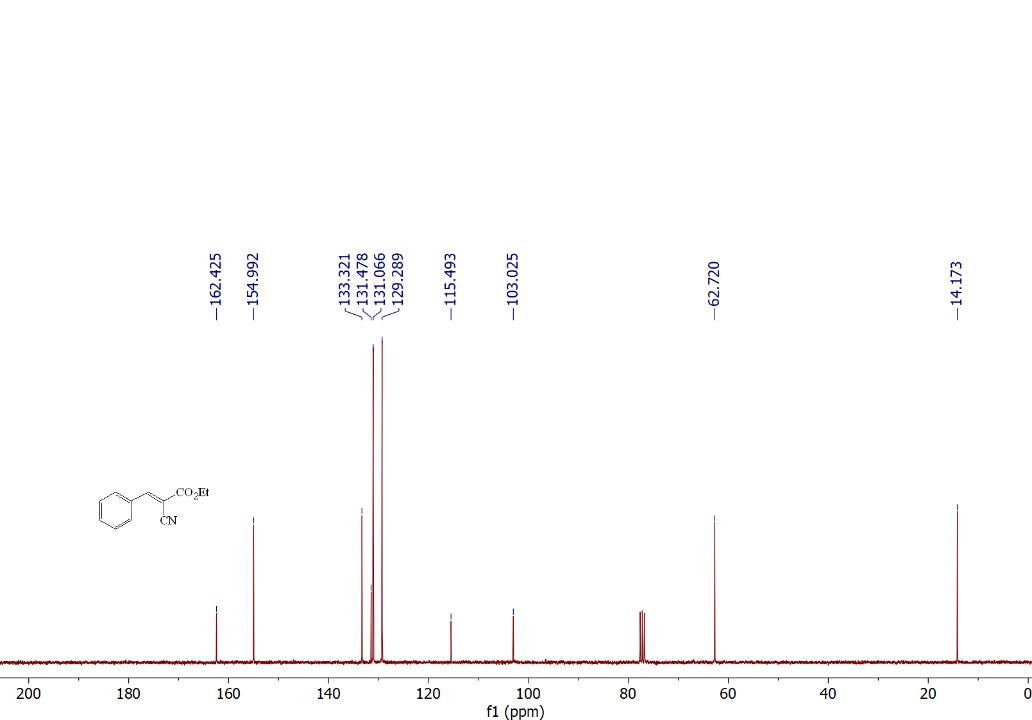
^**

**Figure S16*.*** ^1^H NMR and ^13^C NMR spectra of (E)-ethyl 2-cyano-3-phenylacrylate

^1^HNMR, (300 MHz, CDCl_3_): δ 8.25 (s, 1H), 8.01-7.97 (m, 2H), 7.59-7.47 (m, 3H), 4.38 (q, *J* = 7.2 Hz, 2H), 1.40 (t, *J* = 7.2 Hz, 3H) ppm. ^13^CNMR, (76 MHz, CDCl_3_): δ 162.4, 154.9, 133.3, 131.4, 131.0, 129.2, 115.5, 103.0, 62.7, 14.1 ppm.


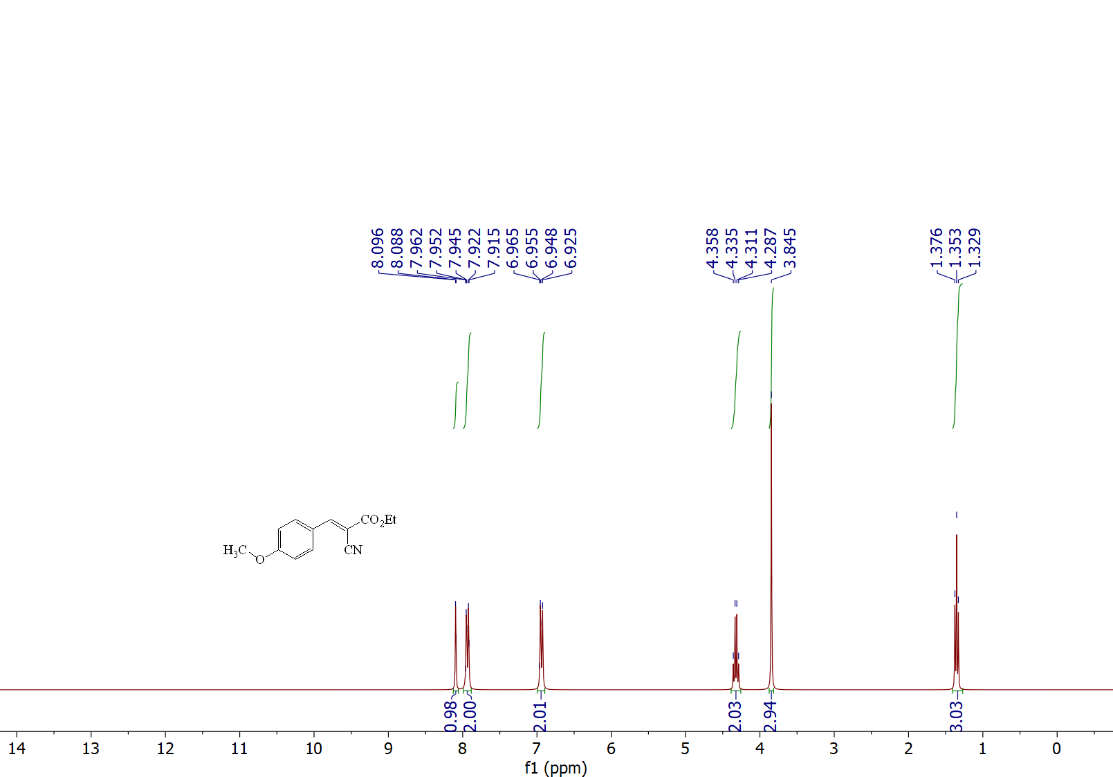


**
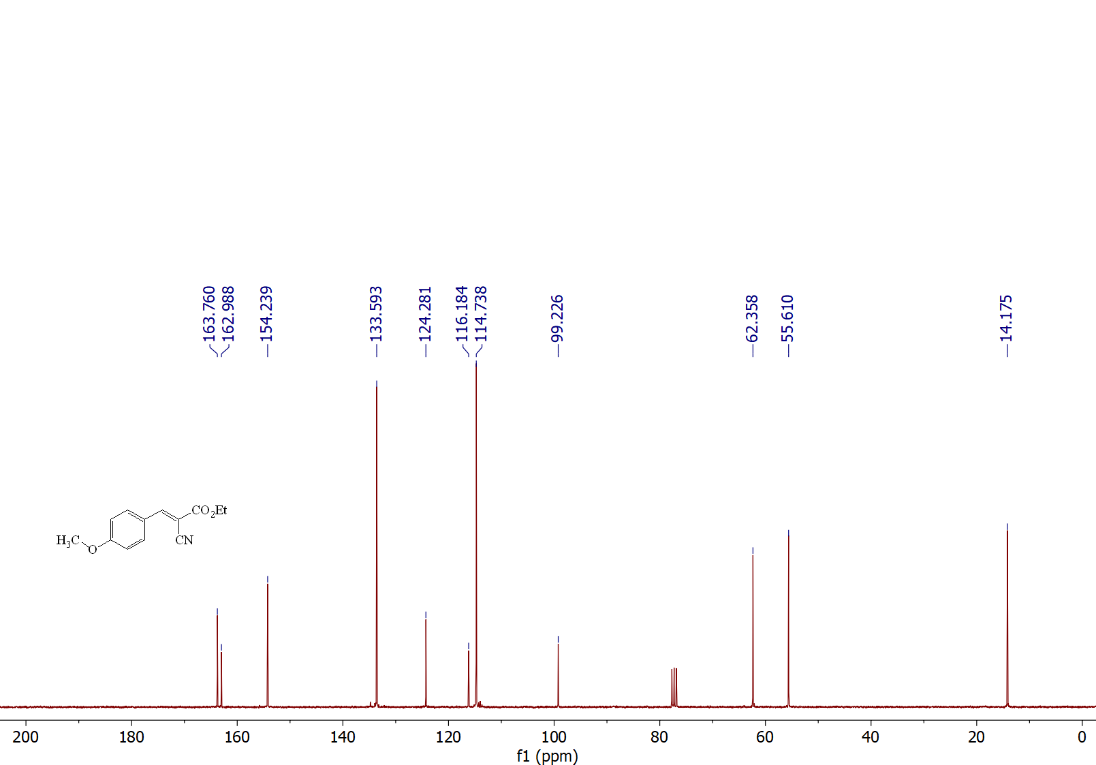
**

**Figure S17*.*** ^1^H NMR and ^13^C NMR spectra of (E)-ethyl 2-cyano-3-(4-methoxyphenyl) acrylate

^1^HNMR (300 MHz, CDCl_3_): δ 8.09 (s, 1 H), 7.96-7.92 (m, 2 H) 6.97-6.93 (m, 2 H), 4.32 (q, *J* = 7.2 Hz, 2 H), 3.84 (s, 3 H), 1.35 (t, *J* = 7.2 Hz, 3 H) ppm. ^13^CNMR, (76 MHz, CDCl_3_): δ 163.8, 163.0, 154.2, 133.6, 124.3, 116.2, 114.7, 99.2, 62.4, 55.6, 14.2 ppm.


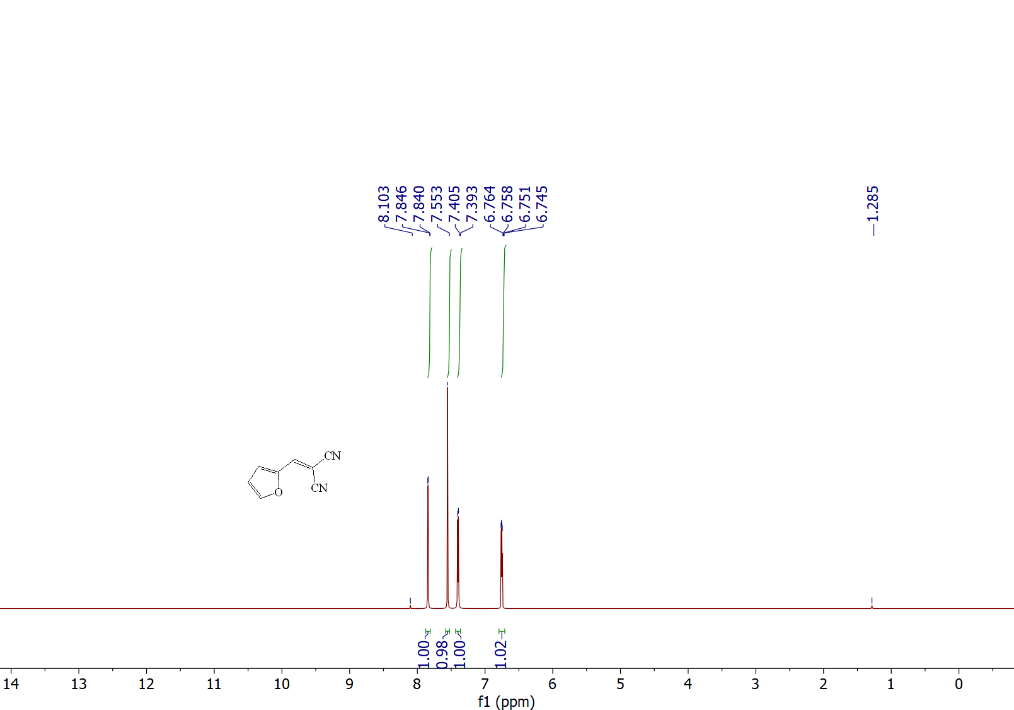


**Figure S18*.*** ^1^H NMR spectrum of (2-furylmethylene) malononitrile

^1^HNMR, (300 MHz, CDCl_3_): δ 7.84 (d, *J* = 1.8 Hz, 1H), 7.55 (s, 1H), 7.40 (d, *J* = 3.6 Hz, 1 H), 6.76-6.74 (m, 1H) ppm.
